# Human infection with a reassortment avian influenza A H3N8 virus: an epidemiological investigation study

**DOI:** 10.1038/s41467-022-34601-1

**Published:** 2022-11-10

**Authors:** Pengtao Bao, Yang Liu, Xiaoai Zhang, Hang Fan, Jie Zhao, Mi Mu, Haiyang Li, Yanhe Wang, Honghan Ge, Shuang Li, Xin Yang, Qianqian Cui, Rui Chen, Liang Gao, Zhihua Sun, Lizhen Gao, Shuang Qiu, Xuchun Liu, Peter W. Horby, Xiubin Li, Liqun Fang, Wei Liu

**Affiliations:** 1 The Eighth Medical Center of Chinese PLA General Hospital, Beijing, 100091 China; 2 Zhumadian Central Hospital, Zhumadian, 463000 China; 3 State Key Laboratory of Pathogen and Biosecurity, Beijing Institute of Microbiology and Epidemiology, Beijing, 100071 China; 4 Zhumadian Second People’s Hospital, Zhumadian, 463000 China; 5 Shangcai Caizhou Hospital, Shangcai County, Zhumadian, 463800 China; 6 Division of HIV/AIDS and Sex-transmitted Virus Vaccines, Institute for Biological Product Control, National Institutes for Food and Drug Control (NIFDC), Beijing, China; 7 Pandemic Sciences Institute, University of Oxford, Oxford, UK; 8 The Third Medical Center of Chinese PLA General Hospital, Beijing, 100039 China; 9 School of Public Health, Anhui Medical University, Hefei, 230032 China

**Keywords:** Influenza virus, Viral infection, Viral epidemiology

## Abstract

A four-year-old boy developed recurrent fever and severe pneumonia in April, 2022. High-throughput sequencing revealed a reassortant avian influenza A-H3N8 virus (A/Henan/ZMD-22-2/2022(H3N8) with avian-origin HA and NA genes. The six internal genes were acquired from Eurasian lineage H9N2 viruses. Molecular substitutions analysis revealed the haemagglutin retained avian-like receptor binding specificity but that PB2 genes possessed sequence changes (E627K) associated with increased virulence and transmissibility in mammalian animal models. The patient developed respiratory failure, liver, renal, coagulation dysfunction and sepsis. Endotracheal intubation and extracorporeal membrane oxygenation were administered. H3N8 RNA was detected from nasopharyngeal swab of a dog, anal swab of a cat, and environmental samples collected in the patient’s house. The full-length HA sequences from the dog and cat were identical to the sequence from the patient. No influenza-like illness was developed and no H3N8 RNA was identified in family members. Serological testing revealed neutralizing antibody response against ZMD-22-2 virus in the patient and three family members. Our results suggest that a triple reassortant H3N8 caused severe human disease. There is some evidence of mammalian adaptation, possible via an intermediary mammalian species, but no evidence of person-to-person transmission. The potential threat from avian influenza viruses warrants continuous evaluation and mitigation.

## Abstract (web summary)

The H3N8 influenza virus is known to circulate in wild birds but cross-species transmission to mammalian hosts was also reported. In this case study, the authors report human infection with a reassortment avian influenza A H3N8 virus in a four-year old boy that developed severe symptoms.

## Introduction

In recent years, many subtypes of avian influenza viruses (AIVs) have been found to be infectious to mammals and to pose a threat to the health of humans and other animals^1–3^. So far, 11 subtypes of AIVs (mainly H5N1, H5N6, H6N1, H7N7, H7N9, H9N2, and H10N8) have been identified to cause human infections. While there are relatively frequent spillover infections (cases of single infections) with avian influenza virus in humans or other mammals^4–7^, only a small proportion of cases have caused epidemics or pandemics in mammalian hosts^8^. AIV infections in humans can result in a wide spectrum of illness, ranging from conjunctivitis and upper respiratory tract disease to pneumonia and multiorgan failure. Low pathogenic avian influenza A (H7N1, H7N2, H7N3, H9N2, or H10N7) virus infections have caused lower respiratory tract illness that is mild (conjunctivitis or uncomplicated influenza-like illness) to moderate in severity^9–11^. Of great concern are the highly pathogenic avian influenza (HPAI) viruses. A (H7) viruses have resulted in conjunctivitis or uncomplicated influenza illness, with only a single fatal case with H7N7 virus infection during an outbreak in the Netherlands^12^. In contrast, HPAI H5N1 and H7N9 virus infections have resulted in case fatality rates of approximately 53% and 34% respectively^13,14^.

The most ubiquitous hemagglutinin (HA) subtype of influenza A viruses is the H3, with a wide host range shown that includes humans, horses, dogs, cats, seals, poultry, pigs, and wild aquatic birds^3,15^. An in vitro experiment also demonstrated its potential to establish successful infections in pigs^16^. However, infection of humans with an H3N8 influenza A virus has not been previously reported. Here we report the human case of infection with H3N8 and describe the epidemiologic, clinical, and genetic features of the case.

## Results

### Clinical features and laboratory abnormalities of the patient

**Fig. 1:**
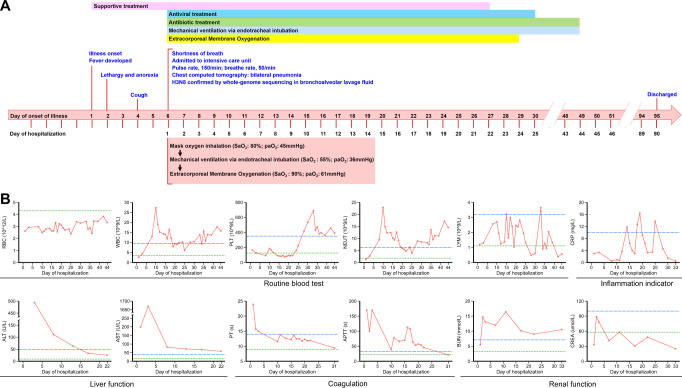
Timeline of the clinical course of the patient, identification of causative pathogen and dynamic changes of laboratory indicators during hospitalization. **A** Timeline of the clinical course of the patient. **B** Dynamic changes of laboratory indicators during hospitalization. The blue dotted line represents the upper limit of the normal range, and the green dotted line represents the lower limit of the normal range. RBC red blood cell, WBC white blood cell, PLT platelet, NEUT neutrophil count, LYM lymphocyte count, CRP C-reactive protein, ALT alanine transaminase, AST aspartate transaminase, PT prothrombin time, APTT activated partial thromboplastin time, BUN blood urea nitrogen, CREA creatinine.

**Fig. 2:**
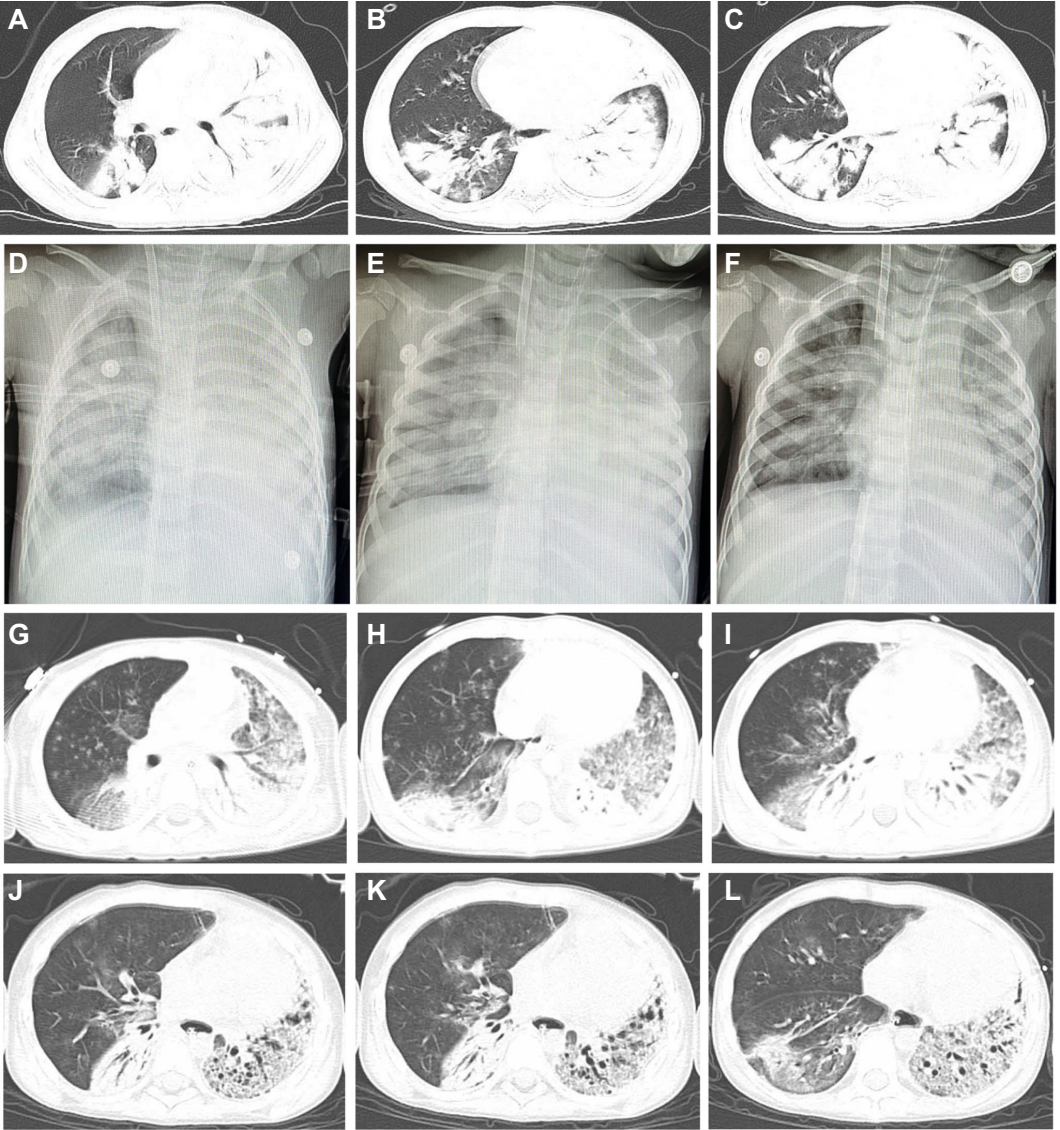
Imaging of the patient’s chest. Computed tomographic (CT) scan obtained on day 6 of illness (**A**–**C**). CT scan showed multiple patchy high-density shadows in both lungs, especially in the lower lobe of the right lung and the left lung. Chest radiographs showed patchy high-density shadows in both lungs on day 7 of illness (**D**). After treatment 2 days, the pulmonary shadows were absorbed slightly on day 8 of illness (**E**) and on day 9 of illness (**F**). The consolidation shadow in the left lung was more absorbed than before on day 16 of illness (**G**–**I**) and day 31 of illness (**J**–**L**).

The patient was a 4-year-old boy living with his father, grandparents, brother and sister, who are otherwise healthy, in Shangcai County, Zhumadian City of Henan province, China (Supplemental Fig. 1). On April 5, 2022, he developed a high fever of 39.3 °C (Fig. 1A), with lethargy and anorexia appearing on April 6, and cough was present on April 8. Although treated with antipyretic drugs and other supportive therapy, his condition did not improve and respiratory symptoms worsened. On April 10, day 6 of illness, he was admitted to a local hospital (Hospital A) for treatment. On admission, he had fever, shortness of breath, and severe respiratory signs and symptoms appeared. The patient was transferred to the intensive care unit in hospital B (Zhumadian Central Hospital, Zhumadian city of Henan). On admission, physical extermination revealed a body temperature of 39.5 °C, moist rales, and cyanosis of lips, accompanied by bilateral axillary lymphadenopathy. His blood pressure was 135/91 mmHg, tachycardic (pulse rate, 150/min), and transcutaneous oxygen saturation level was 80.0 % with mask oxygen inhalation. Blood gas analysis revealed arterial oxygen partial pressure of 45 mmHg. Blood tests showed leukopenia (2.63 10^9^/L), hyperglycemia (blood sugar level 20.3 mmol/L), hyponatremia (129 mmol/L) and hypocalcemia (1.9 mmol/L) (Fig. 1B and Supplemental Fig. 2). There was decreased concentrations of total protein (50.2 g/L), albumin (30.9 g/L) and elevated concentrations of aspartate aminotransferase (AST, 198 U/L), and adenosine deaminase (ADA, 31.0 U/L) (Fig. 1B and Supplemental Fig. 2). Coagulation tests showed a significant prolongation of prothrombin time (PT, 23.9 s), activated partial thromboplastin time (APTT, >170 s), thrombin time (TT, >160 s), and elevated D-dimer (D-D, 5.11 ug/ml) and fibrin degradation products (FDP, 17.36 ug/ml), indicating a coagulation dysfunction (Fig. 1B). NT-proBNP (38.4 pg/ml) and troponin I (<0.012 ng/ml) were normal. High level of lactate dehydrogenase (LDH, 1935 U/L), creatine kinase (CK, 804 U/L) and creatine kinase BB (CK-BB, 55.0 U/L) were observed (Supplemental Fig. 2). A chest computed tomographic (CT) scan showed multiple patchy high-density shadows in both lungs, especially in the lower lobe of the right lung and the left lung (Fig. 2A–C). Antigen test for nine respiratory pathogens were all negative. The bronchoalveolar lavage fluid (BALF) was positive for influenza A by real-time RT-PCR, while negative for the other 13 commonly seen respiratory pathogens. The same sample was simultaneously subject to high throughput sequencing.

Due to high fever of 41 °C, and exacerbation of respiratory symptoms, the patient was further transferred to a tertiary hospital in Zhengzhou City (Hospital C). Laboratory investigations revealed leukopenia, hyperglycemia, hyponatremia, liver dysfunction, renal damage, and coagulation dysfunction, which contributed to multiple organ failure and sepsis (Fig. 1). An inflammatory response characterized by elevated interleukin 6 (IL-6, 13.91 pg/mL), interferon-γ (IFN-γ, 45.55 pg/mL), IL-12P70 (5.63 pg/mL) was observed on day 8 after disease (Supplemental Fig. 2), indicating the development of systemic inflammatory response syndrome. The C-reactive protein (CRP) levels shows modest increases on 14-day hospitalization (11.84 mg/L), and peaked on 19-day hospitalization (16.4 mg/L) (Fig. 1B).

The respiratory symptoms exacerbated with laboratory abnormalities including platelet, albumin, Na^+^ remained abnormally low, while γ-glutamyl transferase (GGT), lactate dehydrogenase (LDH), APTT, FDP, D-D, CK and blood urea nitrogen (BUN) elevated above normal (Fig. 1B, Supplemental Fig. 2). *Pseudomonas aeruginosa* was determined by metagenomic next-generation sequencing in BALF on day 8 of disease. The culture efforts however, yielded no positive result. Extensive treatment, including antiviral therapy (Oseltamivir 30 mg twice a day and interferon α1b 30 ug twice a day), antibiotic therapy (linezolid, meropenem, and compound sulfamethoxazole). After administration of mechanical ventilation via endotracheal intubation on day 6 of illness, the blood oxygen saturation values dropped to 55% and blood gas analysis revealed arterial oxygen partial pressure of 36 mmHg. The patient further received veno-arterial extracorporeal membrane oxygenation (VA-ECMO) on the same day (Supplemental Table 3). Chest radiographs showed patchy high-density shadows in both lungs on day 7 of illness (Fig. 2D). After treatment, the pulmonary shadows slightly diminished (Fig. 2E, F). The consolidation shadow in the left lung gradually diminished on day 16 (Fig. 2G–I) and day 31 of illness (Fig. 2J–L).

His condition gradually improved, with ECMO withdrawn on May 3 and mechanical ventilation ceased on May 23. On June 7, the patient recovered and was discharged after 90-day hospitalization with no sequelae reported.

### Detection and identification of avian influenza A H3N8 virus

By high-throughput sequencing on the BALF collected on April 10, we obtained the whole genome sequence of an influenza A/H3N8 virus (A/Henan/ZMD-22-2/2022(H3N8); GenBank accession number ON342803-ON342810). Phylogenetic analysis revealed ZMD-22-2 as a reassortant H3N8 influenza virus which is distinct from previously reported H3N8 viruses.

**Fig. 3:**
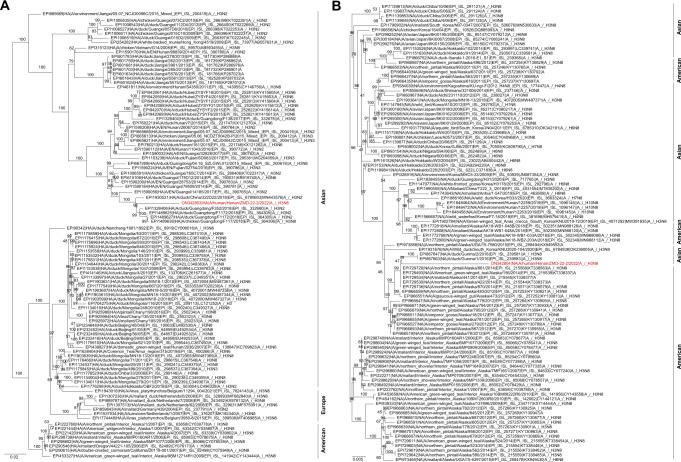
Phylogenetic trees of genes of the A (H3N8) influenza virus. Phylogenetic trees for the full-length HA (**A**) and NA (**B**) genes of H3 and N8 subtype influenza viruses. A/Henan/ZMD-22-2/2022(H3N8) virus was indicated with red color.

**Fig. 4:**
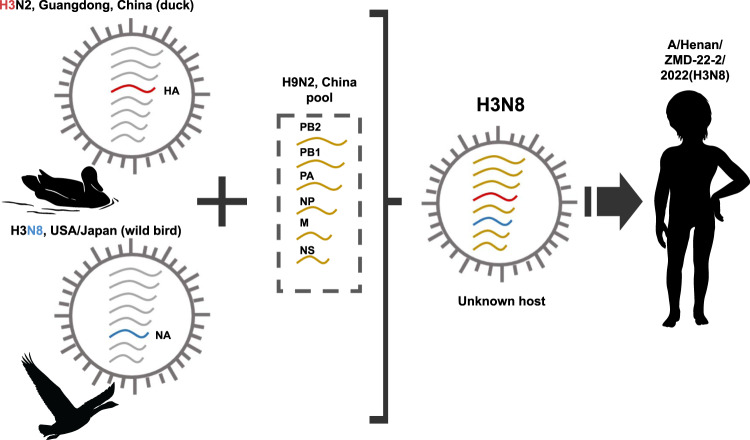
Hypothetical reassortment route and segment origins of the A (H3N8) influenza virus. The colors of the segments indicate their origin.

The most closely related HA gene was in the clade of H3N2 and H3N8 viruses detected in ducks from Guangdong Province (A/duck/Guangdong/F352/2018 (A/H3N2) determined in 2018 with a nucleotide homology of 96.09% (Fig. 3A). The highest similarity of NA segment was to H3N8 viruses in various species of birds in North America in 2014 and Japan in 2016 (A/northern pintail/Alaska/870/2014 (A/H3N8), with nucleotide homologies of 97.07% (Fig. 3B). The nucleotide homologies of internal genes were closely related to H9N2 viruses that had been identified throughout China and isolated from humans, ducks or wild birds in recent years (Supplemental Fig. 3). This indicates that the virus was a reassortant genotype which had undergone complicated mutation and reassortment events (Fig. 4).

The amino acid sequence at cleavage site in HA protein is PEKQTR/GL, indicating that the virus was a low pathogenic avian influenza (LPAI) virus. Genetic features were evaluated based on a well-accepted correlation between receptor binding characteristics and host specificity of influenza viruses^17^, i.e., viruses isolated from wild aquatic birds bind strongly to α-2,3 sialyl glycan (SA 2,3) receptor, in contrast, human-adapted influenza viruses recognize and bind SA 2,6 receptors, which predominate in the human respiratory tract. For the H3N8 virus, the residue 226 and 228 of HA gene were glutamine (Q) and glycine (G), respectively (Supplemental Table 4). The key mutation Q226L and G228S in HA protein which rendered a strong binding to SA 2,6 receptors and increased transmission ability by air, are absent, thus indicating limited ability to bind to cells in the human respiratory tract.

The mutation of E627K was identified in PB2 gene. This mutation had been associated with increased virulence in mice and was reported to be associated with improved replication of avian influenza virus in mammals^17–19^. Besides, the N30D and T215A mutation in M gene and P42S mutation in NS gene were also observed, which mutations were associated with increased virulence in animal models^20,21^.

### Epidemiological investigations

Epidemiological investigation revealed the family had been raising 6 chickens in the back yard, which however, had been killed before the investigation was performed. The patient had intensive contact with a domesticated dog and a cat kept in the household before his illness onset, by feeding and playing with the dog and the cat. Five close-contact family members (grandfather, grandmother, father, eight-year-old brother, and six-year-old sister) who lived together with the patient, and the aunt who had close contact with the patient while he was ill were interviewed. None of them had bred livestock, or reported recent visit to poultry market, the grandfather and brother reported infrequent contact to the dog and cat, although less frequently than the patient. No other significant exposures, i.e., visiting poultry market or exposure to febrile patients were reported. There was a pond 27-m distant from the household of the patient, which wild ducks inhabited, with frequent congregation with the domesticated poultry in the backyard (Supplemental Fig. 1).

The molecular test on the 63 animal and environmental samples yielded 10 positive results for the H3N8, all from the patient’s household, including one nasopharyngeal swab of the dog; one anal swab of the cat; surface swabs of the boarding kennels of cat (1), dog (1), and chicken (1); drinking water (1) and food (1) of the dog; swab of the dining table (1), waste bin (1) and cabinet (1). No positive result was obtained in other 53 animal or environmental samples collected in the household (13) or the village (40).

No obvious illness was observed for the animals, although the dog was less active than usual and labored breathing as recalled by the family. Biochemistry test revealed elevated level of creatine kinase (CK, 323 U/L), lactate dehydrogenase (LDH, 1.29 g/mL) of the dog, Decreased LDH (0.96 g/mL) in the cat (Supplemental Table 5). Nucleotide sequence of full-length HA from the dog and cat were identical to sequence from the patient.

Detection for the H3N8, influenza virus (IFV) A, as well as 13 common respiratory pathogens were all negative for throat swabs collected from the 6 close contacts. The follow-up of the close contacts revealed influenza like illness developed until recently. Serological test on serum of the patient collected on April 10 revealed neutralizing antibody (Nab) titer of 1:121 against ZMD-22-2 virus by using a pseudotyped virus-based assay. Positive Nab was also determined from the grandparents of the patient (1:339 and 1:179, respectively) (Supplemental Table 6).

## Discussion

H3N8 virus is a common subtype of virus detected in wild and domestic ducks^22^, and it has attracted a high attention because of its cross species transmission from avian to mammals^3,23,24^. This represented the identification of human infection with H3N8, since its first identification in Florida in 1963^25^. Although the current H3N8 subtype viruses could infect mammalian hosts, the whole genome sequence showed that it is still avian-like virus. The reassortant among different influenza viruses is considered the main mechanism for the emergence of novel virus, such as H5N1, H7N9, and H10N8 which can adapt to mammals and gain the ability to infect humans. In a likewise manner, a multiple reassortant from different origins was displayed for this H3N8 virus. Genetic evolution of the HA revealed a very close relationship with H3 in ducks that circulated in Guangdong, and the NA gene was derived from birds in both North America and Japan lineages. Still, a high similarity of internal gene with the widely circulating H9N2 in China was demonstrated. The pattern of the Chinese sub-lineage mixing with North America and Japanese influenza viruses remained obscure. It’s speculated that a frequent exchange of influenza virus might have occurred due to wild bird migration, or international poultry trade.

The field investigation revealed a natural environment that was suitable for the congregating and contact among migratory birds with the local backyard poultry, which can promote the transmission of AIV. Geographically, the village was is located in the East Asian-Australian migratory birds’ flyway and it is one of potential stopovers for migratory birds (Supplemental Fig. 1), rendering a logical postulation of this mixture between wild birds and poultry.

It’s notable that H3N8 virus was also detected from the domesticated cat and dog that were in close contact with the patient. Both dogs and cats are known to be susceptible to human influenza and avian influenza strains. Dogs are particularly susceptible to influenza A viruses, including H3N2, H3N8, H5N1, and H6N1.In Asia, respiratory disease caused by influenza virus H3N2 was documented in dogs, and fatal infection with the highly pathogenic avian influenza virus (HPAIV) H5N1 has also been reported^26^. A number of single cases of H5N1 HPAI infections in cats have also been reported in different parts of the world, mainly associated with recent avian outbreaks^27^. Here we observed the H3N8 infected dog developed mild clinical signs, moreover, laboratory abnormality of elevated LDH was observed, possibly indicating a systematic infection. The infected dog excreted virus not only via the respiratory tract but also possibly via the digestive tract as evidenced by positive detection of H3N8 specific RNA in the drinking water. Therefore, both the respiratory and gastrointestinal routes of infection may cause horizontal transmission among dog, cat and the human being. However, it is not possible to infer the direction of transmission, since both dogs and cats are naturally susceptible to influenza virus strains from other hosts, including birds and mammals. Under current situation, both cat and dog are semidomesticated and may highly likely come in contact with wild birds, ducks in the nearby pond, on the other hand, frequently exposed to human and poultry. Transmission of H3N8 virus into dog and cat further to human, or the otherwise manner can both occur. The potential ability of cat and dog to be a “mixing vessel” of diverse origin influenza strains into reassortant might be indicated. Unfortunately, the lack of poultry specimens from the household of the patient, and the unsuccessful sampling of the duck in the neighboring pond means we cannot firmly establish the original zoonotic source of infection nor determine the genetic sequence of the original virus.

There was no evidence of infection in other family members and it is unlikely an outbreaks or epidemics could be induced, due to the biological and ecological barriers of the novel genotype. In line with the epidemiological findings, the genetic characterization revealed that ZMD-22-2 was low pathogenic. On the other hand, the presence of mutations, mainly E627K in PB2 gene and P42S in NS gene, indicated the capacity of this influenza A virus subtype to cross the species barrier, acquire some mammalian adaptations and cause human severe disease.

The patient has developed severe respiratory distress syndrome, yet with no other known respiratory coinfection identified. Although we determined the presence of *Pseudomonas aeruginosa* by high-throughput sequencing, no bacterial culture was obtained, indicating its minor contribution to the overall disease. On the other hand, antibiotic therapy was started empirically on early hospitalization, aimed toward bacterial secondary infection that commonly occurs in the clinical course of severe pneumonia^28^. For patients who present without risk factors or signs of bacterial infection, antibacterial use might be unnecessary.

The study was subject to a major limitation that no virus isolation was performed due to the biosafety concern, thus the antibody test was performed on a pseudovirus system, and the cross reaction with other H3 influenza cannot be excluded. The biological features of the H3N8 need to be further investigated by experimental approaches. Moreover, based on only one case, our understanding of the clinical aspects of H3N8 infection is rather limited. A full understanding of the clinical spectrum might be proposed based on a large-scale population surveillance.

As of preparing the manuscript, an equine influenza (EIV) outbreak caused by H3N8 virus was reported in America^29^. Although not evolved from AIV, it might be transmitted from horses to dogs or birds. It is therefore important to strengthen the surveillance of H3N8 infections among various animal species, based on which a close monitoring of the viral evolution and timely identification of new human cases could be achieved.

## Methods

### Epidemiological investigations and data collection

In April 2022, as part of hospital surveillance of febrile patients in Zhumadian Central Hospital, Henan province, China, a febrile pediatric patient with recurrent fever of unknown cause was screened. Following the identification of infection with an avian influenza virus, an epidemiological investigation was performed on the patient and his close-contact family members and relatives using a standard questionnaire, which included demographic information, pre-existing underlying diseases and the exposure history before the onset of illnesses. To infer the possible infection source of the patient, we performed field investigation in the residence of the patient and the village on April 13. Nasopharyngeal swabs, anal swabs, faecal and blood samples were collected from companion animals, livestock and poultry. The collection of specimens for companion animals, livestock and poultry were conducted by a veterinarian. The environmental samples that included the surface swabs, drinking water, sewage, and water in a pool close to the family were also collected. Throat swabs and blood samples were collected from close contacts of the patient, who were also asked to report for fever (≥38 °C) and influenza-like symptoms for seven days since the interview. All infectious materials were handled in a BSL-2 facility under approved protocols according to Beijing Institute of Microbiology and Epidemiology guidelines. According to the regulations and guidelines of the NHFPC of China, data collection on this patient was part of the routine surveillance and outbreak investigation, and was therefore exempt from the oversight by institutional review board. The patient’s parents gave informed consent. The family members of the patient and all the participating subjects signed consent forms approving the investigation, sample collection and its publication. The procedures were in accordance with the Helsinki declaration of 1975, as revised in 1983.

### Metagenomic analysis and genome assembly

The RNA from BALF of the patient were subject to high-throughput sequencing. Briefly, the Viral RNA was extracted using AllPrep DNA/RNA mini kit (Qiagen, Germany) and sequenced using Illumina Nextseq 550 platform. Sequenced data was assembled with the reference sequences in database using CLC Genomic Workbench v21. The sequence alignment and annotation were performed using CLC Genomic Workbench v21.

### Virus detection

All the collected samples were tested for 14 respiratory pathogens, including IFV A, IFV B, seasonal influenza H3N2 virus, seasonal influenza H1N1 virus, 2009 pandemic influenza A (pH1N1) virus, H7N9 virus, H5N1 virus, respiratory syncytial virus (RSV), human rhinovirus (HRV), human parainfluenza virus (HPIV), human adenovirus (HAdV), human coronavirus (HCoV), human bocavirus (HBoV), and human metapneumovirus (HMPV), by real-time PCR/Reverse Transcriptase-PCR (RT-PCR) (Supplementary Table 1)^30^. Briefly, the nucleic acid was extracted using QIAamp Viral RNA Mini Kit (Qiagen, Germany). For all IFV positive samples, a pair of universal full-length primers (MBTuni-12 and MBTuni-13) were used to reverse transcribe and amplify all eight segments of the virus genome^31^. Specific test for the H3N8 was performed by real-time RT-PCR (Supplementary Table 2). Antigen test for nine respiratory pathogens *(legionella pneumophila, mycoplasma pneumoniae, Coxiella burnetii, chlamydia pneumoniae*, HAdV, RSV, IFV-A, IFV- B, and HPIV) was also performed by using commercial indirect immunofluorescent kit (Vircell SL, Spain) (Supplementary Table 1).

### Cytokine detection

The level of 7 cytokines, IL-4, IL-6, IL-10, IL-17, IL-12p70, tumor necrosis factor-α (TNF-α), and interferon-gamma (IFN-γ), were measured by multiple microsphere flow immunofluorescence methods using the commercial kit (Qingdao Raisecare Biotechnology Co., Ltd, Shandong, China).

### Phylogenetic analysis

The reference nucleotide sequences were downloaded for phylogenetic analysis. Phylogenetic trees were reconstructed based on maximum likelihood method with nucleotide sequences using IQ-TREE (v1.6.12) with GTR + G as the best-fit substitution model and 1000 bootstrap replicates. MAFFT method was used for sequence alignment.

### Pseudovirus based neutralization assay

The H3N8 pseudovirus was generated, based on which the pseudotyped virus neutralization assay was measured by the reduction in Luc gene expression^32^. Briefly, serially diluted samples were incubated with pseudotyped virus in duplicate for 1 h at 37 °C together with the virus control and cell control wells in sextuplicate. Thereafter, 2 × 10^4^ freshly trypsinized Huh7 cells (obtained from Japanese Collection of Research Bioresources) were added to each well of the 96-well plate. After 48 h of incubation at 37 °C with 5% CO_2_, the RLU was measured in accordance with the instruction manual provided by PerkinElmer (Waltham, MA). The ED50 (median effective dilution) was calculated using the Reed-Muench method.

### Reporting summary

Further information on research design is available in the Nature Portfolio Reporting Summary linked to this article.

## Supplementary information

Reporting Summary

Supplementary Information

## Data Availability

The sequence data generated in this study have been deposited in the NCBI GenBank database under accession codes ON342803-ON342810. Relevant data that support the findings of this study is publicly available within the paper and its Supplementary Information Files and Source Data file. Raw data are not publicly available and are protected due to data privacy laws, which were used under license for the current study, but are available upon reasonable request to the corresponding author and with permission from the data provider (Wei Liu). The request will be responded within 1 week. Source data are provided with this paper.

## Source data

Source Data
