# Supplementary material for: Human infection with a reassortment avian influenza A H3N8 virus: an epidemiological investigation study
Source: Nat Commun. 2022 Nov 10;13:6817. doi: 10.1038/s41467-022-34601-1 (PMC9649012; doi:10.1038/s41467-022-34601-1)
Supplement: Supplementary file 2 — Supplementary Information [file 41467_2022_34601_MOESM2_ESM.pdf]

Supplemental Figure 1. The H3N8-infected patient'house and surrounding environment, Zhumadian prefecture, Henan Province, China. A. The location of the patient is within the East Asian-Australian migratory birds' flyway (marked in yellow); B. Schematic of the house of the patient (red contour line) and the neighboring pond (orange contour line); C. The neighboring pond. The companion dog (D) and cat (E) kept in the patient's house**.** Maps were produced using ArcGIS Desktop 10.6.

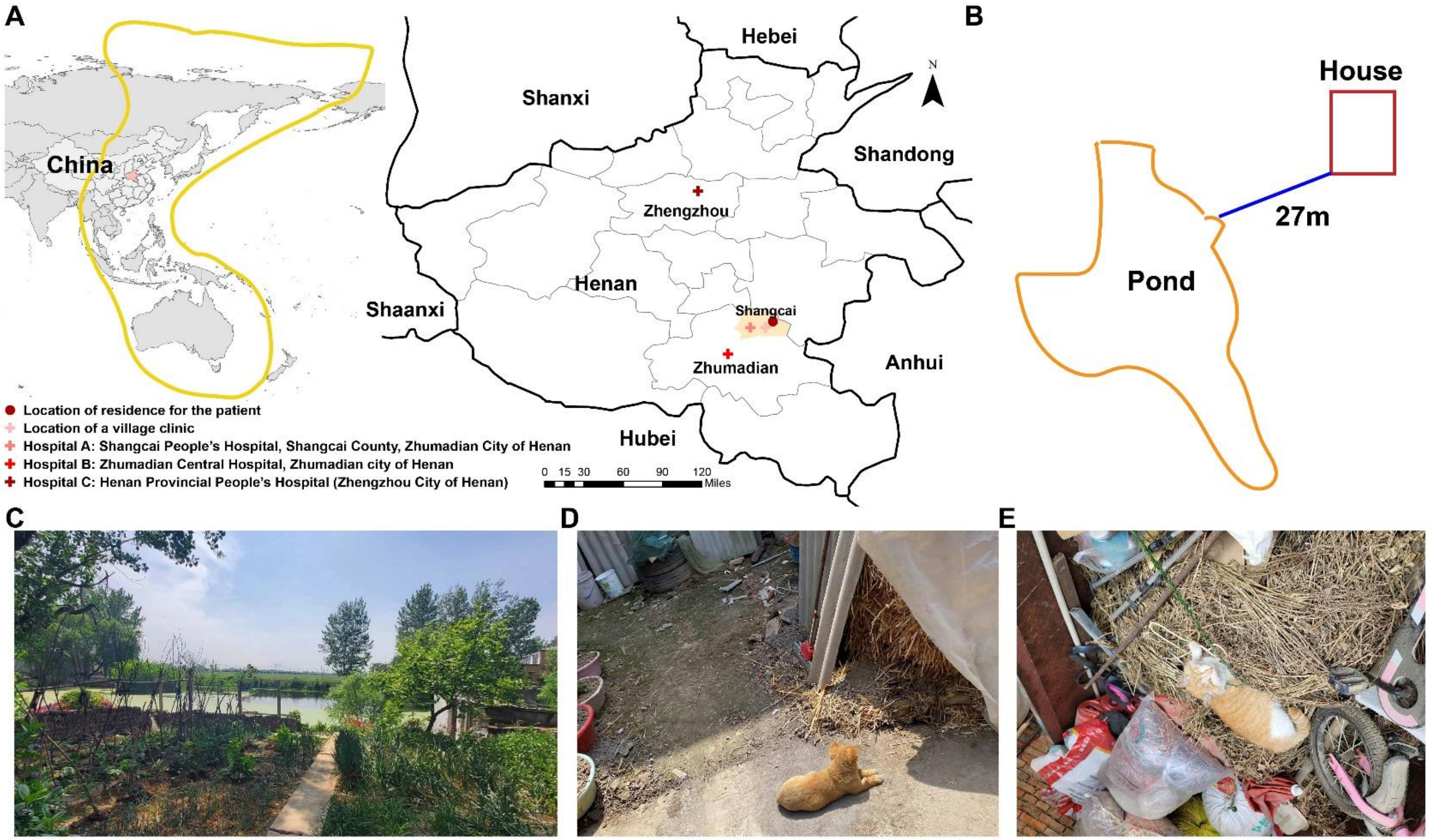

Supplemental Figure 2. Dynamic changes of laboratory indicators during hospitalization. The blue dotted line represents the upper limit of the normal range, and the green dotted line represents the lower limit of the normal range. TP, total protein; ALB, albumin; GGT, gamma-glutamyl transferase; LDH, lactate dehydrogenase; FDP, fibrin degradation products; IL-6, Interleukin 6; IFN-γ, interferon-gamma; IL-12p70, Interleukin 12p70.

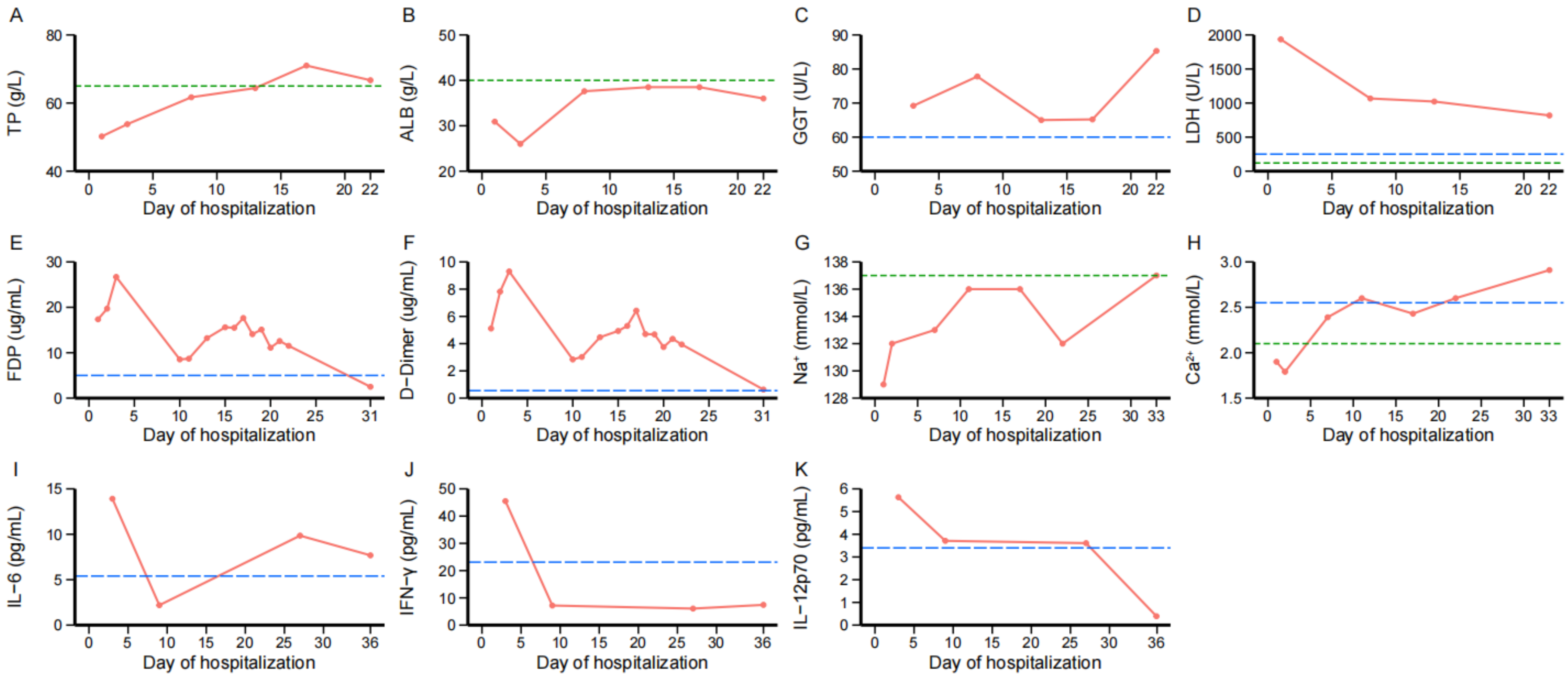

Supplemental Figure 3. Phylogenetic trees of PB2 (A), PB1 (B), PA (C), NP (D), M (E) and NS (F) genes of the novel avian influenza A (H3N8) virus. A/Henan/ZMD-22-2/2022(H3N8) virus was indicated with a red color.

Fig. S3 A(PB2)

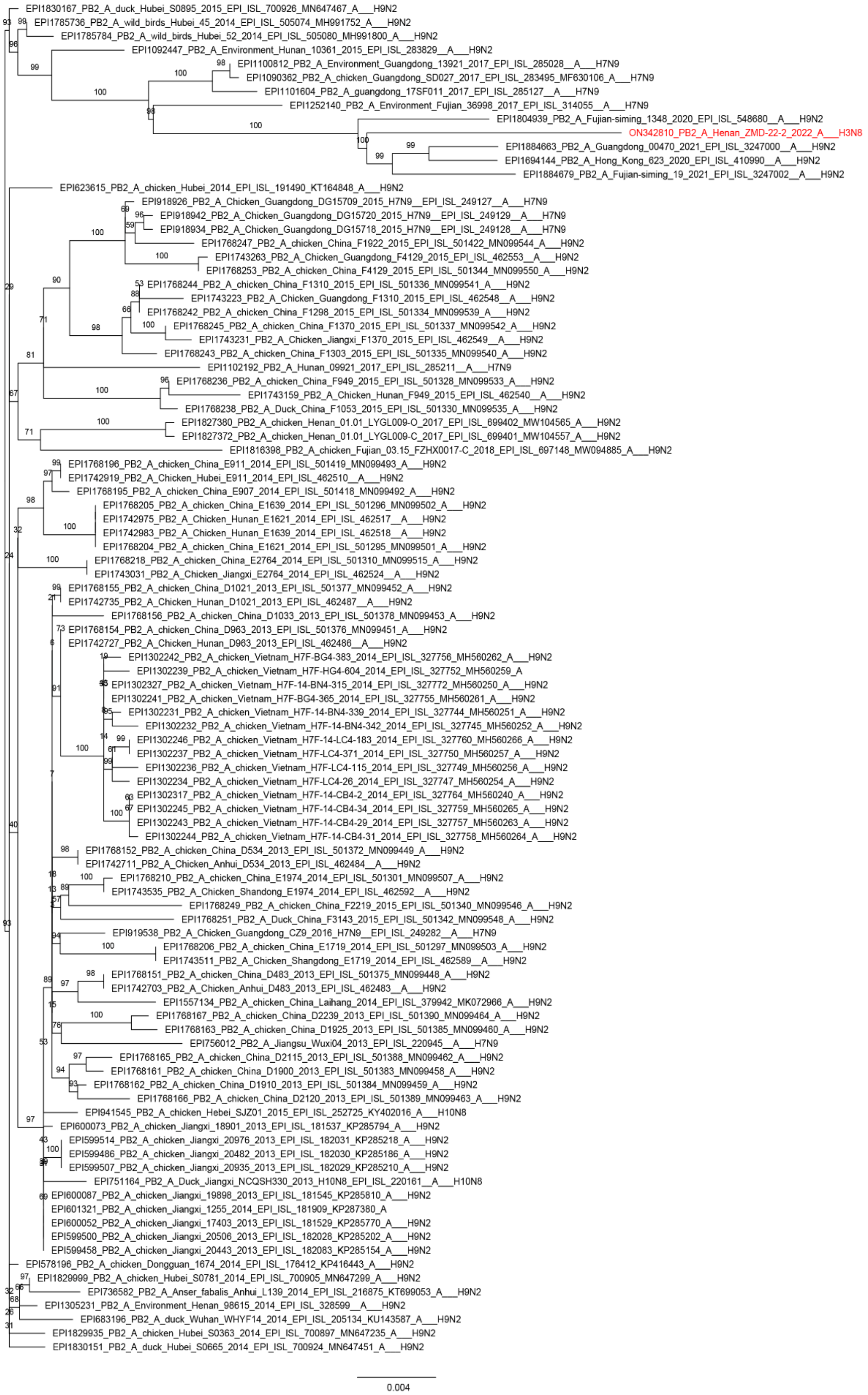

Fig. S3 B(PB1)

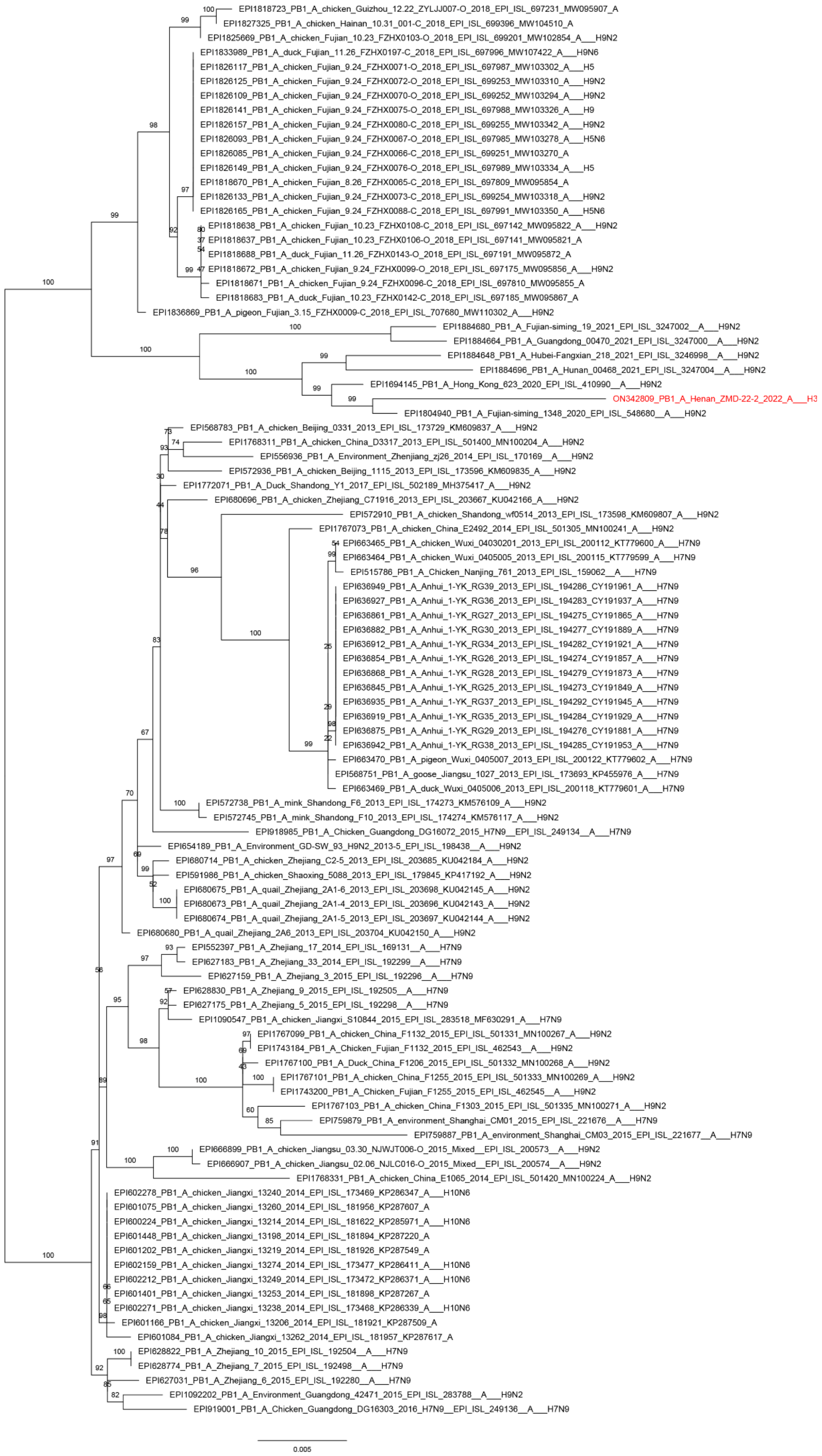
EPI1818723_PB1_A_chicken_Guizhou_12.22_ZYLJJ007-O_2018_EPI_ISL_697231_MW095907_A
EPI1827325_PB1_A_chicken_Hainan_10.31_001-C_2018_EPI_ISL_699396_MW104510_A
EPI1825669_PB1_A_chicken_Fujian_10.23_FZHX0103-O_2018_EPI_ISL_699201_MW102854_A___H9N2
EPI1833989_PB1_A_duck_Fujian_11.26_FZHX0197-C_2018_EPI_ISL_697996_MW107422_A___H9N6
EPI1826117_PB1_A_chicken_Fujian_9.24_FZHX0071-O_2018_EPI_ISL_697987_MW103302_A___H5
EPI1826125_PB1_A_chicken_Fujian_9.24_FZHX0072-O_2018_EPI_ISL_699253_MW103310_A___H9N2
EPI1826109_PB1_A_chicken_Fujian_9.24_FZHX0070-O_2018_EPI_ISL_699252_MW103294_A___H9N2
EPI1826141_PB1_A_chicken_Fujian_9.24_FZHX0075-O_2018_EPI_ISL_697988_MW103326_A___H9
EPI1826157_PB1_A_chicken_Fujian_9.24_FZHX0080-C_2018_EPI_ISL_699255_MW103342_A___H9N2
EPI1826093_PB1_A_chicken_Fujian_9.24_FZHX0067-O_2018_EPI_ISL_697985_MW103278_A___H5N6
EPI1826085_PB1_A_chicken_Fujian_9.24_FZHX0066-C_2018_EPI_ISL_699251_MW103270_A
EPI1826149_PB1_A_chicken_Fujian_9.24_FZHX0076-O_2018_EPI_ISL_697989_MW103334_A___H5
EPI1818670_PB1_A_chicken_Fujian_8.26_FZHX0065-C_2018_EPI_ISL_697809_MW095854_A
EPI1826133_PB1_A_chicken_Fujian_9.24_FZHX0073-C_2018_EPI_ISL_699254_MW103318_A___H9N2
EPI1826165_PB1_A_chicken_Fujian_9.24_FZHX0088-C_2018_EPI_ISL_697991_MW103350_A___H5N6
EPI1818638_PB1_A_chicken_Fujian_10.23_FZHX0108-C_2018_EPI_ISL_697142_MW095822_A___H9N2
EPI1818637_PB1_A_chicken_Fujian_10.23_FZHX0106-O_2018_EPI_ISL_697141_MW095821_A
EPI1818688_PB1_A_duck_Fujian_11.26_FZHX0143-O_2018_EPI_ISL_697191_MW095872_A
EPI1818672_PB1_A_chicken_Fujian_9.24_FZHX0099-O_2018_EPI_ISL_697175_MW095856_A___H9N2
EPI1818671_PB1_A_chicken_Fujian_9.24_FZHX0096-C_2018_EPI_ISL_697810_MW095855_A
EPI1818683_PB1_A_duck_Fujian_10.23_FZHX0142-C_2018_EPI_ISL_697185_MW095867_A
EPI1836869_PB1_A_pigeon_Fujian_3.15_FZHX0009-C_2018_EPI_ISL_707680_MW110302_A___H9N2
EPI1884680_PB1_A_Fujian-siming_19_2021_EPI_ISL_3247002__A___H9N2
EPI1884664_PB1_A_Guangdong_00470_2021_EPI_ISL_3247000__A___H9N2
EPI1884648_PB1_A_Hubei-Fangxian_218_2021_EPI_ISL_3246998__A___H9N2
EPI1884696_PB1_A_Hunan_00468_2021_EPI_ISL_3247004__A___H9N2
EPI1694145_PB1_A_Hong_Kong_623_2020_EPI_ISL_410990__A___H9N2
ON342809_PB1_A_Henan_ZMD-22-2_2022_A___H3
EPI1804940_PB1_A_Fujian-siming_1348_2020_EPI_ISL_548680__A___H9N2
EPI568783_PB1_A_chicken_Beijing_0331_2013_EPI_ISL_173729_KM609837_A___H9N2
EPI1768311_PB1_A_chicken_China_D3317_2013_EPI_ISL_501400_MN100204_A___H9N2
EPI556936_PB1_A_Environment_Zhenjiang_zj26_2014_EPI_ISL_170169__A___H9N2
EPI572936_PB1_A_chicken_Beijing_1115_2013_EPI_ISL_173596_KM609835_A___H9N2
EPI1772071_PB1_A_Duck_Shandong_Y1_2017_EPI_ISL_502189_MH375417_A___H9N2
EPI680696_PB1_A_chicken_Zhejiang_C71916_2013_EPI_ISL_203667_KU042166_A___H9N2
EPI572910_PB1_A_chicken_Shandong_wf0514_2013_EPI_ISL_173598_KM609807_A___H9N2
EPI1767073_PB1_A_chicken_China_E2492_2014_EPI_ISL_501305_MN100241_A___H9N2
EPI663465_PB1_A_chicken_Wuxi_04030201_2013_EPI_ISL_200112_KT779600_A___H7N9
EPI663464_PB1_A_chicken_Wuxi_0405005_2013_EPI_ISL_200115_KT779599_A___H7N9
EPI515786_PB1_A_Chicken_Nanjing_761_2013_EPI_ISL_159062__A___H7N9
EPI636949_PB1_A_Anhui_1-YK_RG39_2013_EPI_ISL_194286_CY191961_A___H7N9
EPI636927_PB1_A_Anhui_1-YK_RG36_2013_EPI_ISL_194283_CY191937_A___H7N9
EPI636861_PB1_A_Anhui_1-YK_RG27_2013_EPI_ISL_194275_CY191865_A___H7N9
EPI636882_PB1_A_Anhui_1-YK_RG30_2013_EPI_ISL_194277_CY191889_A___H7N9
EPI636912_PB1_A_Anhui_1-YK_RG34_2013_EPI_ISL_194282_CY191921_A___H7N9
EPI636854_PB1_A_Anhui_1-YK_RG26_2013_EPI_ISL_194274_CY191857_A___H7N9
EPI636868_PB1_A_Anhui_1-YK_RG28_2013_EPI_ISL_194279_CY191873_A___H7N9
EPI636845_PB1_A_Anhui_1-YK_RG25_2013_EPI_ISL_194273_CY191849_A___H7N9
EPI636935_PB1_A_Anhui_1-YK_RG37_2013_EPI_ISL_194292_CY191945_A___H7N9
EPI636919_PB1_A_Anhui_1-YK_RG35_2013_EPI_ISL_194284_CY191929_A___H7N9
EPI636875_PB1_A_Anhui_1-YK_RG29_2013_EPI_ISL_194276_CY191881_A___H7N9
EPI636942_PB1_A_Anhui_1-YK_RG38_2013_EPI_ISL_194285_CY191953_A___H7N9
EPI663470_PB1_A_pigeon_Wuxi_0405007_2013_EPI_ISL_200122_KT779602_A___H7N9
EPI568751_PB1_A_goose_Jiangsu_1027_2013_EPI_ISL_173693_KP455976_A___H7N9
EPI663469_PB1_A_duck_Wuxi_0405006_2013_EPI_ISL_200118_KT779601_A___H7N9
EPI572738_PB1_A_mink_Shandong_F6_2013_EPI_ISL_174273_KM576109_A___H9N2
EPI572745_PB1_A_mink_Shandong_F10_2013_EPI_ISL_174274_KM576117_A___H9N2
EPI918985_PB1_A_Chicken_Guangdong_DG16072_2015_H7N9__EPI_ISL_249134__A___H7N9
EPI654189_PB1_A_Environment_GD-SW_93_H9N2_2013-5_EPI_ISL_198438__A___H9N2
EPI680714_PB1_A_chicken_Zhejiang_C2-5_2013_EPI_ISL_203685_KU042184_A___H9N2
EPI591988_PB1_A_chicken_Shaoxing_5088_2013_EPI_ISL_179845_KP417192_A___H9N2
EPI680675_PB1_A_quail_Zhejiang_2A1-6_2013_EPI_ISL_203698_KU042145_A___H9N2
EPI680673_PB1_A_quail_Zhejiang_2A1-4_2013_EPI_ISL_203696_KU042143_A___H9N2
EPI680674_PB1_A_quail_Zhejiang_2A1-5_2013_EPI_ISL_203697_KU042144_A___H9N2
EPI680680_PB1_A_quail_Zhejiang_2A6_2013_EPI_ISL_203704_KU042150_A___H9N2
EPI552397_PB1_A_Zhejiang_17_2014_EPI_ISL_169131__A___H7N9
EPI627183_PB1_A_Zhejiang_33_2014_EPI_ISL_192299__A___H7N9
EPI627159_PB1_A_Zhejiang_3_2015_EPI_ISL_192296__A___H7N9
EPI628830_PB1_A_Zhejiang_9_2015_EPI_ISL_192505__A___H7N9
EPI627175_PB1_A_Zhejiang_5_2015_EPI_ISL_192298__A___H7N9
EPI1090547_PB1_A_chicken_Jiangxi_S10844_2015_EPI_ISL_283518_MF630291_A___H7N9
EPI1767099_PB1_A_chicken_China_F1132_2015_EPI_ISL_501331_MN100267_A___H9N2
EPI1743184_PB1_A_Chicken_Fujian_F1132_2015_EPI_ISL_462543__A___H9N2
EPI1767100_PB1_A_Duck_China_F1206_2015_EPI_ISL_501332_MN100268_A___H9N2
EPI1767101_PB1_A_chicken_China_F1255_2015_EPI_ISL_501333_MN100269_A___H9N2
EPI1743200_PB1_A_Chicken_Fujian_F1255_2015_EPI_ISL_462545__A___H9N2
EPI1767103_PB1_A_chicken_China_F1303_2015_EPI_ISL_501335_MN100271_A___H9N2
EPI759879_PB1_A_environment_Shanghai_CM01_2015_EPI_ISL_221676__A___H7N9
EPI759887_PB1_A_environment_Shanghai_CM03_2015_EPI_ISL_221677__A___H7N9
EPI666899_PB1_A_chicken_Jiangsu_03.30_NJWJT006-O_2015_Mixed__EPI_ISL_200573__A___H9N2
EPI666907_PB1_A_chicken_Jiangsu_02.06_NJLC016-O_2015_Mixed__EPI_ISL_200574__A___H9N2
EPI1768331_PB1_A_chicken_China_E1065_2014_EPI_ISL_501420_MN100224_A___H9N2
EPI602278_PB1_A_chicken_Jiangxi_13240_2014_EPI_ISL_173469_KP286347_A___H10N6
EPI601075_PB1_A_chicken_Jiangxi_13260_2014_EPI_ISL_181956_KP287607_A
EPI600224_PB1_A_chicken_Jiangxi_13214_2014_EPI_ISL_181622_KP285971_A___H10N6
EPI601448_PB1_A_chicken_Jiangxi_13198_2014_EPI_ISL_181894_KP287220_A
EPI601202_PB1_A_chicken_Jiangxi_13219_2014_EPI_ISL_181926_KP287549_A
EPI602159_PB1_A_chicken_Jiangxi_13274_2014_EPI_ISL_173477_KP286411_A___H10N6
EPI602212_PB1_A_chicken_Jiangxi_13249_2014_EPI_ISL_173472_KP286371_A___H10N6
EPI601401_PB1_A_chicken_Jiangxi_13253_2014_EPI_ISL_181898_KP287267_A
EPI602271_PB1_A_chicken_Jiangxi_13238_2014_EPI_ISL_173468_KP286339_A___H10N6
EPI601166_PB1_A_chicken_Jiangxi_13206_2014_EPI_ISL_181921_KP287509_A
EPI601084_PB1_A_chicken_Jiangxi_13262_2014_EPI_ISL_181957_KP287617_A
EPI628822_PB1_A_Zhejiang_10_2015_EPI_ISL_192504__A___H7N9
EPI628774_PB1_A_Zhejiang_7_2015_EPI_ISL_192498__A___H7N9
EPI627031_PB1_A_Zhejiang_6_2015_EPI_ISL_192280__A___H7N9
EPI1092202_PB1_A_Environment_Guangdong_42471_2015_EPI_ISL_283788__A___H9N2
EPI919001_PB1_A_Chicken_Guangdong_DG16303_2016_H7N9__EPI_ISL_249136__A___H7N9
0.005

Fig. S3 C(PA)

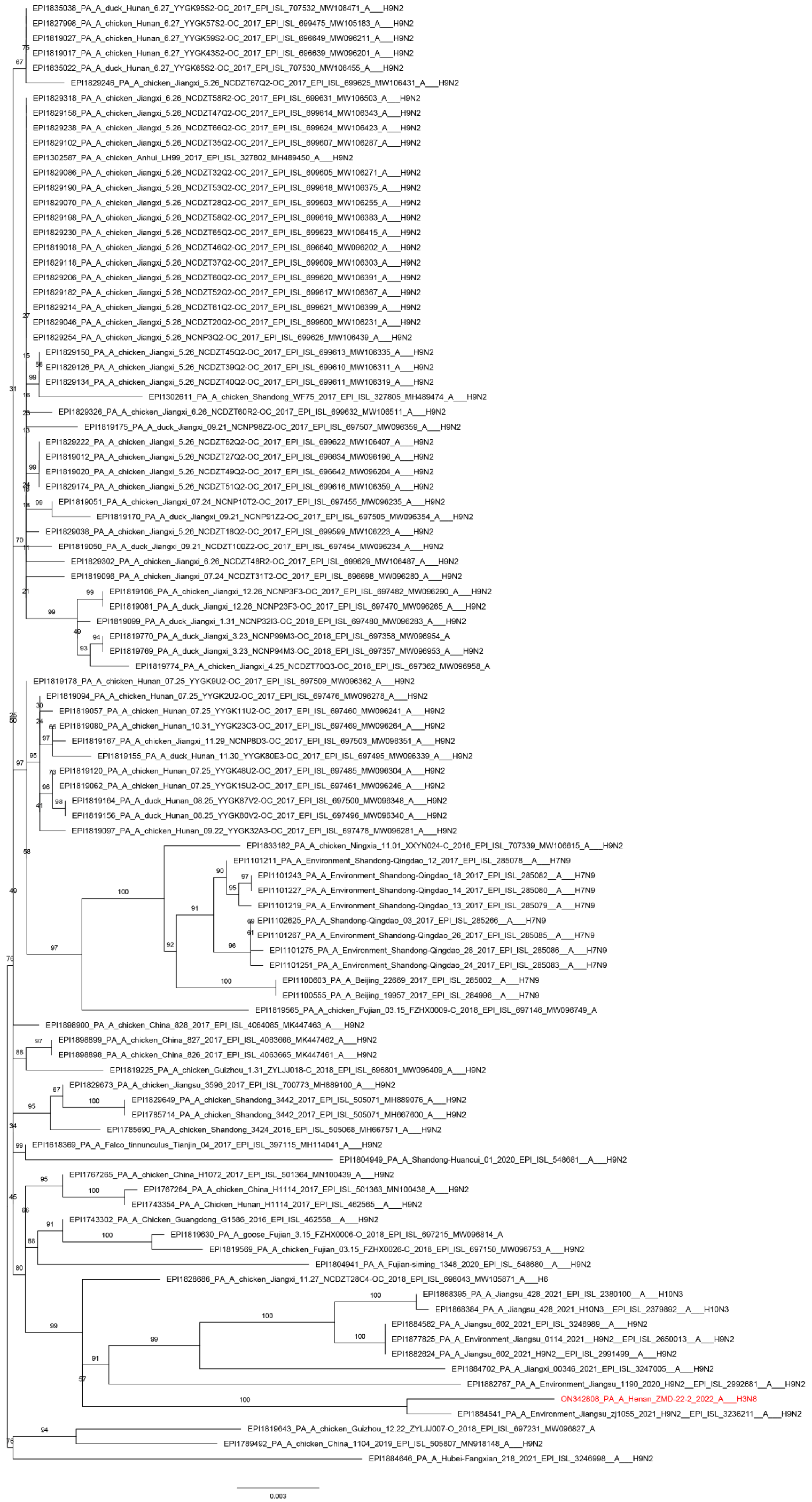

EPI1835038_PA_A_duck_Hunan_6.27_YYGK95S2-OC_2017_EPI_ISL_707532_MW108471_A___H9N2
EPI1827998_PA_A_chicken_Hunan_6.27_YYGK57S2-OC_2017_EPI_ISL_699475_MW105183_A___H9N2
EPI1819027_PA_A_chicken_Hunan_6.27_YYGK59S2-OC_2017_EPI_ISL_696649_MW096211_A___H9N2
EPI1819017_PA_A_chicken_Hunan_6.27_YYGK43S2-OC_2017_EPI_ISL_696639_MW096201_A___H9N2
EPI1835022_PA_A_duck_Hunan_6.27_YYGK65S2-OC_2017_EPI_ISL_707530_MW108455_A___H9N2
EPI1829246_PA_A_chicken_Jiangxi_5.26_NCDZT67Q2-OC_2017_EPI_ISL_699625_MW106431_A___H9N2
EPI1829318_PA_A_chicken_Jiangxi_6.26_NCDZT58R2-OC_2017_EPI_ISL_699631_MW106503_A___H9N2
EPI1829158_PA_A_chicken_Jiangxi_5.26_NCDZT47Q2-OC_2017_EPI_ISL_699614_MW106343_A___H9N2
EPI1829238_PA_A_chicken_Jiangxi_5.26_NCDZT66Q2-OC_2017_EPI_ISL_699624_MW106423_A___H9N2
EPI1829102_PA_A_chicken_Jiangxi_5.26_NCDZT35Q2-OC_2017_EPI_ISL_699607_MW106287_A___H9N2
EPI1302587_PA_A_chicken_Anhui_LH99_2017_EPI_ISL_327802_MH489450_A___H9N2
EPI1829086_PA_A_chicken_Jiangxi_5.26_NCDZT32Q2-OC_2017_EPI_ISL_699605_MW106271_A___H9N2
EPI1829190_PA_A_chicken_Jiangxi_5.26_NCDZT53Q2-OC_2017_EPI_ISL_699618_MW106375_A___H9N2
EPI1829070_PA_A_chicken_Jiangxi_5.26_NCDZT28Q2-OC_2017_EPI_ISL_699603_MW106255_A___H9N2
EPI1829198_PA_A_chicken_Jiangxi_5.26_NCDZT58Q2-OC_2017_EPI_ISL_699619_MW106383_A___H9N2
EPI1829230_PA_A_chicken_Jiangxi_5.26_NCDZT65Q2-OC_2017_EPI_ISL_699623_MW106415_A___H9N2
EPI1819018_PA_A_chicken_Jiangxi_5.26_NCDZT46Q2-OC_2017_EPI_ISL_696640_MW096202_A___H9N2
EPI1829118_PA_A_chicken_Jiangxi_5.26_NCDZT37Q2-OC_2017_EPI_ISL_699609_MW106303_A___H9N2
EPI1829206_PA_A_chicken_Jiangxi_5.26_NCDZT60Q2-OC_2017_EPI_ISL_699620_MW106391_A___H9N2
EPI1829182_PA_A_chicken_Jiangxi_5.26_NCDZT52Q2-OC_2017_EPI_ISL_699617_MW106367_A___H9N2
EPI1829214_PA_A_chicken_Jiangxi_5.26_NCDZT61Q2-OC_2017_EPI_ISL_699621_MW106399_A___H9N2
EPI1829046_PA_A_chicken_Jiangxi_5.26_NCDZT20Q2-OC_2017_EPI_ISL_699600_MW106231_A___H9N2
EPI1829254_PA_A_chicken_Jiangxi_5.26_NCNP3Q2-OC_2017_EPI_ISL_699626_MW106439_A___H9N2
EPI1829150_PA_A_chicken_Jiangxi_5.26_NCDZT45Q2-OC_2017_EPI_ISL_699613_MW106335_A___H9N2
EPI1829126_PA_A_chicken_Jiangxi_5.26_NCDZT39Q2-OC_2017_EPI_ISL_699610_MW106311_A___H9N2
EPI1829134_PA_A_chicken_Jiangxi_5.26_NCDZT40Q2-OC_2017_EPI_ISL_699611_MW106319_A___H9N2
EPI1302611_PA_A_chicken_Shandong_WF75_2017_EPI_ISL_327805_MH489474_A___H9N2
EPI1829326_PA_A_chicken_Jiangxi_6.26_NCDZT60R2-OC_2017_EPI_ISL_699632_MW106511_A___H9N2
EPI1819175_PA_A_duck_Jiangxi_09.21_NCNP98Z2-OC_2017_EPI_ISL_697507_MW096359_A___H9N2
EPI1829222_PA_A_chicken_Jiangxi_5.26_NCDZT62Q2-OC_2017_EPI_ISL_699622_MW106407_A___H9N2
EPI1819012_PA_A_chicken_Jiangxi_5.26_NCDZT27Q2-OC_2017_EPI_ISL_696634_MW096196_A___H9N2
EPI1819020_PA_A_chicken_Jiangxi_5.26_NCDZT49Q2-OC_2017_EPI_ISL_696642_MW096204_A___H9N2
EPI1829174_PA_A_chicken_Jiangxi_5.26_NCDZT51Q2-OC_2017_EPI_ISL_699616_MW106359_A___H9N2
EPI1819051_PA_A_chicken_Jiangxi_07.24_NCNP10T2-OC_2017_EPI_ISL_697455_MW096235_A___H9N2
EPI1819170_PA_A_duck_Jiangxi_09.21_NCNP91Z2-OC_2017_EPI_ISL_697505_MW096354_A___H9N2
EPI1829038_PA_A_chicken_Jiangxi_5.26_NCDZT18Q2-OC_2017_EPI_ISL_699599_MW106223_A___H9N2
EPI1819050_PA_A_duck_Jiangxi_09.21_NCDZT100Z2-OC_2017_EPI_ISL_697454_MW096234_A___H9N2
EPI1829302_PA_A_chicken_Jiangxi_6.26_NCDZT48R2-OC_2017_EPI_ISL_699629_MW106487_A___H9N2
EPI1819096_PA_A_chicken_Jiangxi_07.24_NCDZT31T2-OC_2017_EPI_ISL_696698_MW096280_A___H9N2
EPI1819106_PA_A_chicken_Jiangxi_12.26_NCNP3F3-OC_2017_EPI_ISL_697482_MW096290_A___H9N2
EPI1819081_PA_A_duck_Jiangxi_12.26_NCNP23F3-OC_2017_EPI_ISL_697470_MW096265_A___H9N2
EPI1819099_PA_A_duck_Jiangxi_1.31_NCNP32I3-OC_2018_EPI_ISL_697480_MW096283_A___H9N2
EPI1819770_PA_A_duck_Jiangxi_3.23_NCNP99M3-OC_2018_EPI_ISL_697358_MW096954_A
EPI1819769_PA_A_duck_Jiangxi_3.23_NCNP94M3-OC_2018_EPI_ISL_697357_MW096953_A___H9N2
EPI1819774_PA_A_chicken_Jiangxi_4.25_NCDZT70Q3-OC_2018_EPI_ISL_697362_MW096958_A
EPI1819178_PA_A_chicken_Hunan_07.25_YYGK9U2-OC_2017_EPI_ISL_697509_MW096362_A___H9N2
EPI1819094_PA_A_chicken_Hunan_07.25_YYGK2U2-OC_2017_EPI_ISL_697476_MW096278_A___H9N2
EPI1819057_PA_A_chicken_Hunan_07.25_YYGK11U2-OC_2017_EPI_ISL_697460_MW096241_A___H9N2
EPI1819080_PA_A_chicken_Hunan_10.31_YYGK23C3-OC_2017_EPI_ISL_697469_MW096264_A___H9N2
EPI1819167_PA_A_chicken_Jiangxi_11.29_NCNP8D3-OC_2017_EPI_ISL_697503_MW096351_A___H9N2
EPI1819155_PA_A_duck_Hunan_11.30_YYGK80E3-OC_2017_EPI_ISL_697495_MW096339_A___H9N2
EPI1819120_PA_A_chicken_Hunan_07.25_YYGK48U2-OC_2017_EPI_ISL_697485_MW096304_A___H9N2
EPI1819062_PA_A_chicken_Hunan_07.25_YYGK15U2-OC_2017_EPI_ISL_697461_MW096246_A___H9N2
EPI1819164_PA_A_duck_Hunan_08.25_YYGK87V2-OC_2017_EPI_ISL_697500_MW096348_A___H9N2
EPI1819156_PA_A_duck_Hunan_08.25_YYGK80V2-OC_2017_EPI_ISL_697496_MW096340_A___H9N2
EPI1819097_PA_A_chicken_Hunan_09.22_YYGK32A3-OC_2017_EPI_ISL_697478_MW096281_A___H9N2
EPI1833182_PA_A_chicken_Ningxia_11.01_XXYN024-C_2016_EPI_ISL_707339_MW106615_A___H9N2
EPI1101211_PA_A_Environment_Shandong-Qingdao_12_2017_EPI_ISL_285078__A___H7N9
EPI1101243_PA_A_Environment_Shandong-Qingdao_18_2017_EPI_ISL_285082__A___H7N9
EPI1101227_PA_A_Environment_Shandong-Qingdao_14_2017_EPI_ISL_285080__A___H7N9
EPI1101219_PA_A_Environment_Shandong-Qingdao_13_2017_EPI_ISL_285079__A___H7N9
EPI1102625_PA_A_Shandong-Qingdao_03_2017_EPI_ISL_285266__A___H7N9
EPI1101267_PA_A_Environment_Shandong-Qingdao_26_2017_EPI_ISL_285085__A___H7N9
EPI1101275_PA_A_Environment_Shandong-Qingdao_28_2017_EPI_ISL_285086__A___H7N9
EPI1101251_PA_A_Environment_Shandong-Qingdao_24_2017_EPI_ISL_285083__A___H7N9
EPI1100603_PA_A_Beijing_22669_2017_EPI_ISL_285002__A___H7N9
EPI1100555_PA_A_Beijing_19957_2017_EPI_ISL_284996__A___H7N9
EPI1819565_PA_A_chicken_Fujian_03.15_FZHX0009-C_2018_EPI_ISL_697146_MW096749_A
EPI1898900_PA_A_chicken_China_828_2017_EPI_ISL_4064085_MK447463_A___H9N2
EPI1898899_PA_A_chicken_China_827_2017_EPI_ISL_4063666_MK447462_A___H9N2
EPI1898898_PA_A_chicken_China_826_2017_EPI_ISL_4063665_MK447461_A___H9N2
EPI1819225_PA_A_chicken_Guizhou_1.31_ZYLJJ018-C_2018_EPI_ISL_696801_MW096409_A___H9N2
EPI1829673_PA_A_chicken_Jiangsu_3596_2017_EPI_ISL_700773_MH889100_A___H9N2
EPI1829649_PA_A_chicken_Shandong_3442_2017_EPI_ISL_505071_MH889076_A___H9N2
EPI1785714_PA_A_chicken_Shandong_3442_2017_EPI_ISL_505071_MH667600_A___H9N2
EPI1785690_PA_A_chicken_Shandong_3424_2016_EPI_ISL_505068_MH667571_A___H9N2
EPI1618369_PA_A_Falco_tinnunculus_Tianjin_04_2017_EPI_ISL_397115_MH114041_A___H9N2
EPI1804949_PA_A_Shandong-Huancui_01_2020_EPI_ISL_548681__A___H9N2
EPI1767265_PA_A_chicken_China_H1072_2017_EPI_ISL_501364_MN100439_A___H9N2
EPI1767264_PA_A_chicken_China_H1114_2017_EPI_ISL_501363_MN100438_A___H9N2
EPI1743354_PA_A_Chicken_Hunan_H1114_2017_EPI_ISL_462565__A___H9N2
EPI1743302_PA_A_Chicken_Guangdong_G1586_2016_EPI_ISL_462558__A___H9N2
EPI1819630_PA_A_goose_Fujian_3.15_FZHX0006-O_2018_EPI_ISL_697215_MW096814_A
EPI1819569_PA_A_chicken_Fujian_03.15_FZHX0026-C_2018_EPI_ISL_697150_MW096753_A___H9N2
EPI1804941_PA_A_Fujian-siming_1348_2020_EPI_ISL_548680__A___H9N2
EPI1828686_PA_A_chicken_Jiangxi_11.27_NCDZT28C4-OC_2018_EPI_ISL_698043_MW105871_A___H6
EPI1868395_PA_A_Jiangsu_428_2021_EPI_ISL_2380100__A___H10N3
EPI1868384_PA_A_Jiangsu_428_2021_H10N3__EPI_ISL_2379892__A___H10N3
EPI1884582_PA_A_Jiangsu_602_2021_EPI_ISL_3246989__A___H9N2
EPI1877825_PA_A_Environment_Jiangsu_0114_2021__H9N2__EPI_ISL_2650013__A___H9N2
EPI1882624_PA_A_Jiangsu_602_2021_H9N2__EPI_ISL_2991499__A___H9N2
EPI1884702_PA_A_Jiangxi_00346_2021_EPI_ISL_3247005__A___H9N2
EPI1882767_PA_A_Environment_Jiangsu_1190_2020_H9N2__EPI_ISL_2992681__A___H9N2
ON342808_PA_A_Henan_ZMD-22-2_2022_A___H3N8
EPI1884541_PA_A_Environment_Jiangsu_zj1055_2021_H9N2__EPI_ISL_3236211__A___H9N2
EPI1819643_PA_A_chicken_Guizhou_12.22_ZYLJJ007-O_2018_EPI_ISL_697231_MW096827_A
EPI1789492_PA_A_chicken_China_1104_2019_EPI_ISL_505807_MN918148_A___H9N2
EPI1884646_PA_A_Hubei-Fangxian_218_2021_EPI_ISL_3246998__A___H9N2
0.003

Fig. S3 D(NP)

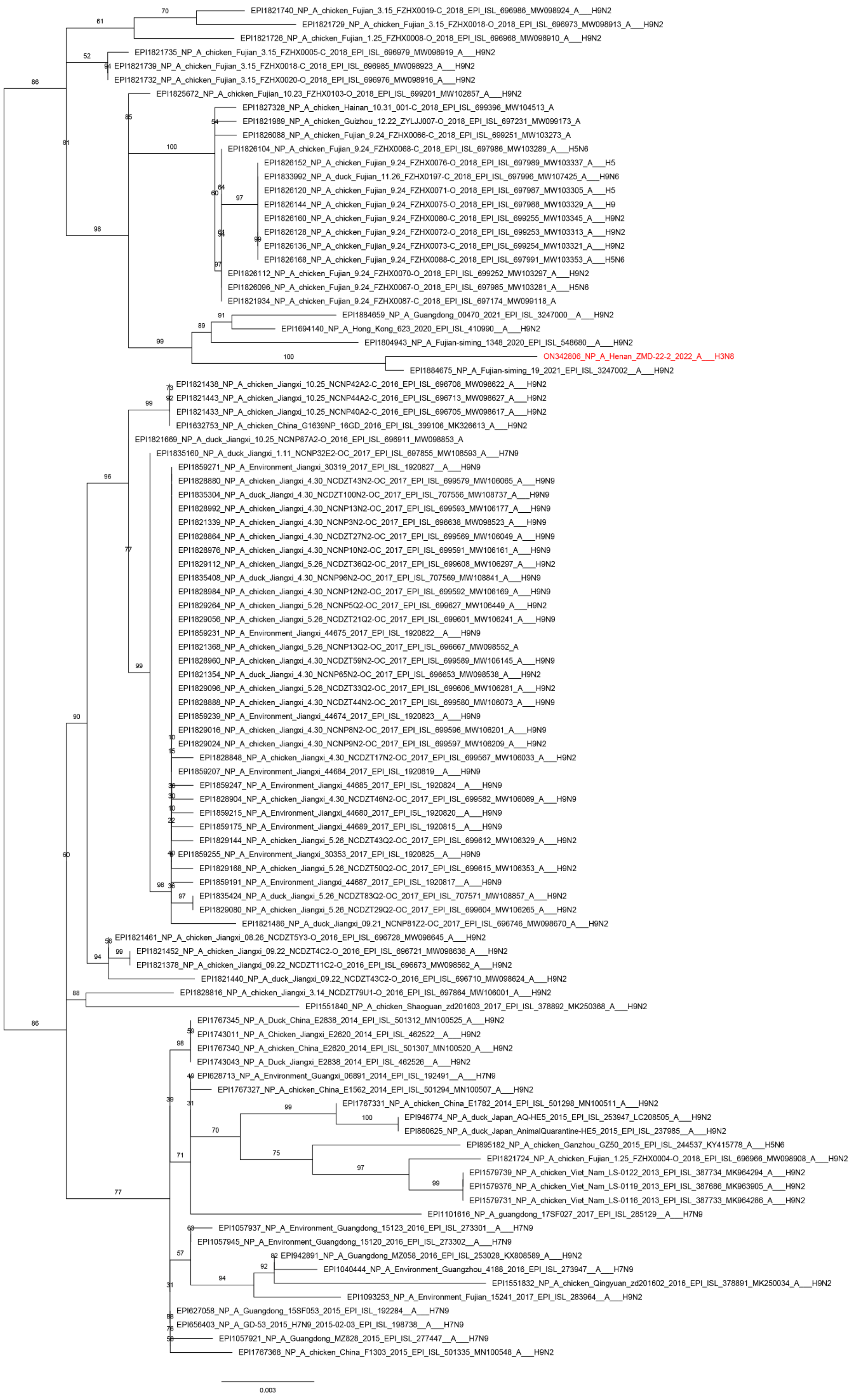

EPI1821740_NP_A_chicken_Fujian_3.15_FZHX0019-C_2018_EPI_ISL_696986_MW098924_A___H9N2
EPI1821729_NP_A_chicken_Fujian_3.15_FZHX0018-O_2018_EPI_ISL_696973_MW098913_A___H9N2
EPI1821726_NP_A_chicken_Fujian_1.25_FZHX0008-O_2018_EPI_ISL_696968_MW098910_A___H9N2
EPI1821735_NP_A_chicken_Fujian_3.15_FZHX0005-C_2018_EPI_ISL_696979_MW098919_A___H9N2
EPI1821739_NP_A_chicken_Fujian_3.15_FZHX0018-C_2018_EPI_ISL_696985_MW098923_A___H9N2
EPI1821732_NP_A_chicken_Fujian_3.15_FZHX0020-O_2018_EPI_ISL_696976_MW098916_A___H9N2
EPI1825672_NP_A_chicken_Fujian_10.23_FZHX0103-O_2018_EPI_ISL_699201_MW102857_A___H9N2
EPI1827328_NP_A_chicken_Hainan_10.31_001-C_2018_EPI_ISL_699396_MW104513_A
EPI1821989_NP_A_chicken_Guizhou_12.22_ZYLJJ007-O_2018_EPI_ISL_697231_MW099173_A
EPI1826088_NP_A_chicken_Fujian_9.24_FZHX0066-C_2018_EPI_ISL_699251_MW103273_A
EPI1826104_NP_A_chicken_Fujian_9.24_FZHX0068-C_2018_EPI_ISL_697986_MW103289_A___H5N6
EPI1826152_NP_A_chicken_Fujian_9.24_FZHX0076-O_2018_EPI_ISL_697989_MW103337_A___H5
EPI1833992_NP_A_duck_Fujian_11.26_FZHX0197-C_2018_EPI_ISL_697996_MW107425_A___H9N6
EPI1826120_NP_A_chicken_Fujian_9.24_FZHX0071-O_2018_EPI_ISL_697987_MW103305_A___H5
EPI1826144_NP_A_chicken_Fujian_9.24_FZHX0075-O_2018_EPI_ISL_697988_MW103329_A___H9
EPI1826160_NP_A_chicken_Fujian_9.24_FZHX0080-C_2018_EPI_ISL_699255_MW103345_A___H9N2
EPI1826128_NP_A_chicken_Fujian_9.24_FZHX0072-O_2018_EPI_ISL_699253_MW103313_A___H9N2
EPI1826136_NP_A_chicken_Fujian_9.24_FZHX0073-C_2018_EPI_ISL_699254_MW103321_A___H9N2
EPI1826168_NP_A_chicken_Fujian_9.24_FZHX0088-C_2018_EPI_ISL_697991_MW103353_A___H5N6
EPI1826112_NP_A_chicken_Fujian_9.24_FZHX0070-O_2018_EPI_ISL_699252_MW103297_A___H9N2
EPI1826096_NP_A_chicken_Fujian_9.24_FZHX0067-O_2018_EPI_ISL_697985_MW103281_A___H5N6
EPI1821934_NP_A_chicken_Fujian_9.24_FZHX0087-C_2018_EPI_ISL_697174_MW099118_A
EPI1884659_NP_A_Guangdong_00470_2021_EPI_ISL_3247000__A___H9N2
EPI1694140_NP_A_Hong_Kong_623_2020_EPI_ISL_410990__A___H9N2
EPI1804943_NP_A_Fujian-siming_1348_2020_EPI_ISL_548680__A___H9N2
ON342806_NP_A_Henan_ZMD-22-2_2022_A___H3N8
EPI1884675_NP_A_Fujian-siming_19_2021_EPI_ISL_3247002__A___H9N2
EPI1821438_NP_A_chicken_Jiangxi_10.25_NCNP42A2-C_2016_EPI_ISL_696708_MW098622_A___H9N2
EPI1821443_NP_A_chicken_Jiangxi_10.25_NCNP44A2-C_2016_EPI_ISL_696713_MW098627_A___H9N2
EPI1821433_NP_A_chicken_Jiangxi_10.25_NCNP40A2-C_2016_EPI_ISL_696705_MW098617_A___H9N2
EPI1632753_NP_A_chicken_China_G1639NP_16GD_2016_EPI_ISL_399106_MK326613_A___H9N2
EPI1821669_NP_A_duck_Jiangxi_10.25_NCNP87A2-O_2016_EPI_ISL_696911_MW098853_A
EPI1835160_NP_A_duck_Jiangxi_1.11_NCNP32E2-OC_2017_EPI_ISL_697855_MW108593_A___H7N9
EPI1859271_NP_A_Environment_Jiangxi_30319_2017_EPI_ISL_1920827__A___H9N9
EPI1828880_NP_A_chicken_Jiangxi_4.30_NCDZT43N2-OC_2017_EPI_ISL_699579_MW106065_A___H9N9
EPI1835304_NP_A_duck_Jiangxi_4.30_NCDZT100N2-OC_2017_EPI_ISL_707556_MW108737_A___H9N9
EPI1828992_NP_A_chicken_Jiangxi_4.30_NCNP13N2-OC_2017_EPI_ISL_699593_MW106177_A___H9N9
EPI1821339_NP_A_chicken_Jiangxi_4.30_NCNP3N2-OC_2017_EPI_ISL_696638_MW098523_A___H9N9
EPI1828864_NP_A_chicken_Jiangxi_4.30_NCDZT27N2-OC_2017_EPI_ISL_699569_MW106049_A___H9N9
EPI1828976_NP_A_chicken_Jiangxi_4.30_NCNP10N2-OC_2017_EPI_ISL_699591_MW106161_A___H9N9
EPI1829112_NP_A_chicken_Jiangxi_5.26_NCDZT36Q2-OC_2017_EPI_ISL_699608_MW106297_A___H9N2
EPI1835408_NP_A_duck_Jiangxi_4.30_NCNP96N2-OC_2017_EPI_ISL_707569_MW108841_A___H9N9
EPI1828984_NP_A_chicken_Jiangxi_4.30_NCNP12N2-OC_2017_EPI_ISL_699592_MW106169_A___H9N9
EPI1829264_NP_A_chicken_Jiangxi_5.26_NCNP5Q2-OC_2017_EPI_ISL_699627_MW106449_A___H9N2
EPI1829056_NP_A_chicken_Jiangxi_5.26_NCDZT21Q2-OC_2017_EPI_ISL_699601_MW106241_A___H9N9
EPI1859231_NP_A_Environment_Jiangxi_44675_2017_EPI_ISL_1920822__A___H9N9
EPI1821368_NP_A_chicken_Jiangxi_5.26_NCNP13Q2-OC_2017_EPI_ISL_696667_MW098552_A
EPI1828960_NP_A_chicken_Jiangxi_4.30_NCDZT59N2-OC_2017_EPI_ISL_699589_MW106145_A___H9N9
EPI1821354_NP_A_duck_Jiangxi_4.30_NCNP65N2-OC_2017_EPI_ISL_696653_MW098538_A___H9N2
EPI1829096_NP_A_chicken_Jiangxi_5.26_NCDZT33Q2-OC_2017_EPI_ISL_699606_MW106281_A___H9N2
EPI1828888_NP_A_chicken_Jiangxi_4.30_NCDZT44N2-OC_2017_EPI_ISL_699580_MW106073_A___H9N9
EPI1859239_NP_A_Environment_Jiangxi_44674_2017_EPI_ISL_1920823__A___H9N9
EPI1829016_NP_A_chicken_Jiangxi_4.30_NCNP8N2-OC_2017_EPI_ISL_699596_MW106201_A___H9N9
EPI1829024_NP_A_chicken_Jiangxi_4.30_NCNP9N2-OC_2017_EPI_ISL_699597_MW106209_A___H9N2
EPI1828848_NP_A_chicken_Jiangxi_4.30_NCDZT17N2-OC_2017_EPI_ISL_699567_MW106033_A___H9N2
EPI1859207_NP_A_Environment_Jiangxi_44684_2017_EPI_ISL_1920819__A___H9N9
EPI1859247_NP_A_Environment_Jiangxi_44685_2017_EPI_ISL_1920824__A___H9N9
EPI1828904_NP_A_chicken_Jiangxi_4.30_NCDZT46N2-OC_2017_EPI_ISL_699582_MW106089_A___H9N9
EPI1859215_NP_A_Environment_Jiangxi_44680_2017_EPI_ISL_1920820__A___H9N9
EPI1859175_NP_A_Environment_Jiangxi_44689_2017_EPI_ISL_1920815__A___H9N9
EPI1829144_NP_A_chicken_Jiangxi_5.26_NCDZT43Q2-OC_2017_EPI_ISL_699612_MW106329_A___H9N2
EPI1859255_NP_A_Environment_Jiangxi_30353_2017_EPI_ISL_1920825__A___H9N9
EPI1829168_NP_A_chicken_Jiangxi_5.26_NCDZT50Q2-OC_2017_EPI_ISL_699615_MW106353_A___H9N2
EPI1859191_NP_A_Environment_Jiangxi_44687_2017_EPI_ISL_1920817__A___H9N9
EPI1835424_NP_A_duck_Jiangxi_5.26_NCDZT83Q2-OC_2017_EPI_ISL_707571_MW108857_A___H9N2
EPI1829080_NP_A_chicken_Jiangxi_5.26_NCDZT29Q2-OC_2017_EPI_ISL_699604_MW106265_A___H9N2
EPI1821486_NP_A_duck_Jiangxi_09.21_NCNP81Z2-OC_2017_EPI_ISL_696746_MW098670_A___H9N2
EPI1821461_NP_A_chicken_Jiangxi_08.26_NCDZT5Y3-O_2016_EPI_ISL_696728_MW098645_A___H9N2
EPI1821452_NP_A_chicken_Jiangxi_09.22_NCDZT4C2-O_2016_EPI_ISL_696721_MW098636_A___H9N2
EPI1821378_NP_A_chicken_Jiangxi_09.22_NCDZT11C2-O_2016_EPI_ISL_696673_MW098562_A___H9N2
EPI1821440_NP_A_duck_Jiangxi_09.22_NCDZT43C2-O_2016_EPI_ISL_696710_MW098624_A___H9N2
EPI1828816_NP_A_chicken_Jiangxi_3.14_NCDZT79U1-O_2016_EPI_ISL_697864_MW106001_A___H9N2
EPI1551840_NP_A_chicken_Shaoguan_zd201603_2017_EPI_ISL_378892_MK250368_A___H9N2
EPI1767345_NP_A_Duck_China_E2838_2014_EPI_ISL_501312_MN100525_A___H9N2
EPI1743011_NP_A_Chicken_Jiangxi_E2620_2014_EPI_ISL_462522__A___H9N2
EPI1767340_NP_A_chicken_China_E2620_2014_EPI_ISL_501307_MN100520_A___H9N2
EPI1743043_NP_A_Duck_Jiangxi_E2838_2014_EPI_ISL_462526__A___H9N2
EPI628713_NP_A_Environment_Guangxi_06891_2014_EPI_ISL_192491__A___H7N9
EPI1767327_NP_A_chicken_China_E1562_2014_EPI_ISL_501294_MN100507_A___H9N2
EPI1767331_NP_A_chicken_China_E1782_2014_EPI_ISL_501298_MN100511_A___H9N2
EPI946774_NP_A_duck_Japan_AQ-HE5_2015_EPI_ISL_253947_LC208505_A___H9N2
EPI860625_NP_A_duck_Japan_AnimalQuarantine-HE5_2015_EPI_ISL_237985__A___H9N2
EPI895182_NP_A_chicken_Ganzhou_GZ50_2015_EPI_ISL_244537_KY415778_A___H5N6
EPI1821724_NP_A_chicken_Fujian_1.25_FZHX0004-O_2018_EPI_ISL_696966_MW098908_A___H9N2
EPI1579739_NP_A_chicken_Viet_Nam_LS-0122_2013_EPI_ISL_387734_MK964294_A___H9N2
EPI1579376_NP_A_chicken_Viet_Nam_LS-0119_2013_EPI_ISL_387686_MK963905_A___H9N2
EPI1579731_NP_A_chicken_Viet_Nam_LS-0116_2013_EPI_ISL_387733_MK964286_A___H9N2
EPI1101616_NP_A_guangdong_17SF027_2017_EPI_ISL_285129__A___H7N9
EPI1057937_NP_A_Environment_Guangdong_15123_2016_EPI_ISL_273301__A___H7N9
EPI1057945_NP_A_Environment_Guangdong_15120_2016_EPI_ISL_273302__A___H7N9
EPI942891_NP_A_Guangdong_MZ058_2016_EPI_ISL_253028_KX808589_A___H9N2
EPI1040444_NP_A_Environment_Guangzhou_4188_2016_EPI_ISL_273947__A___H7N9
EPI1551832_NP_A_chicken_Qingyuan_zd201602_2016_EPI_ISL_378891_MK250034_A___H9N2
EPI1093253_NP_A_Environment_Fujian_15241_2017_EPI_ISL_283964__A___H9N2
EPI627058_NP_A_Guangdong_15SF053_2015_EPI_ISL_192284__A___H7N9
EPI656403_NP_A_GD-53_2015_H7N9_2015-02-03_EPI_ISL_198738__A___H7N9
EPI1057921_NP_A_Guangdong_MZ828_2015_EPI_ISL_277447__A___H7N9
EPI1767368_NP_A_chicken_China_F1303_2015_EPI_ISL_501335_MN100548_A___H9N2
0.003

Fig. S3 E(M)

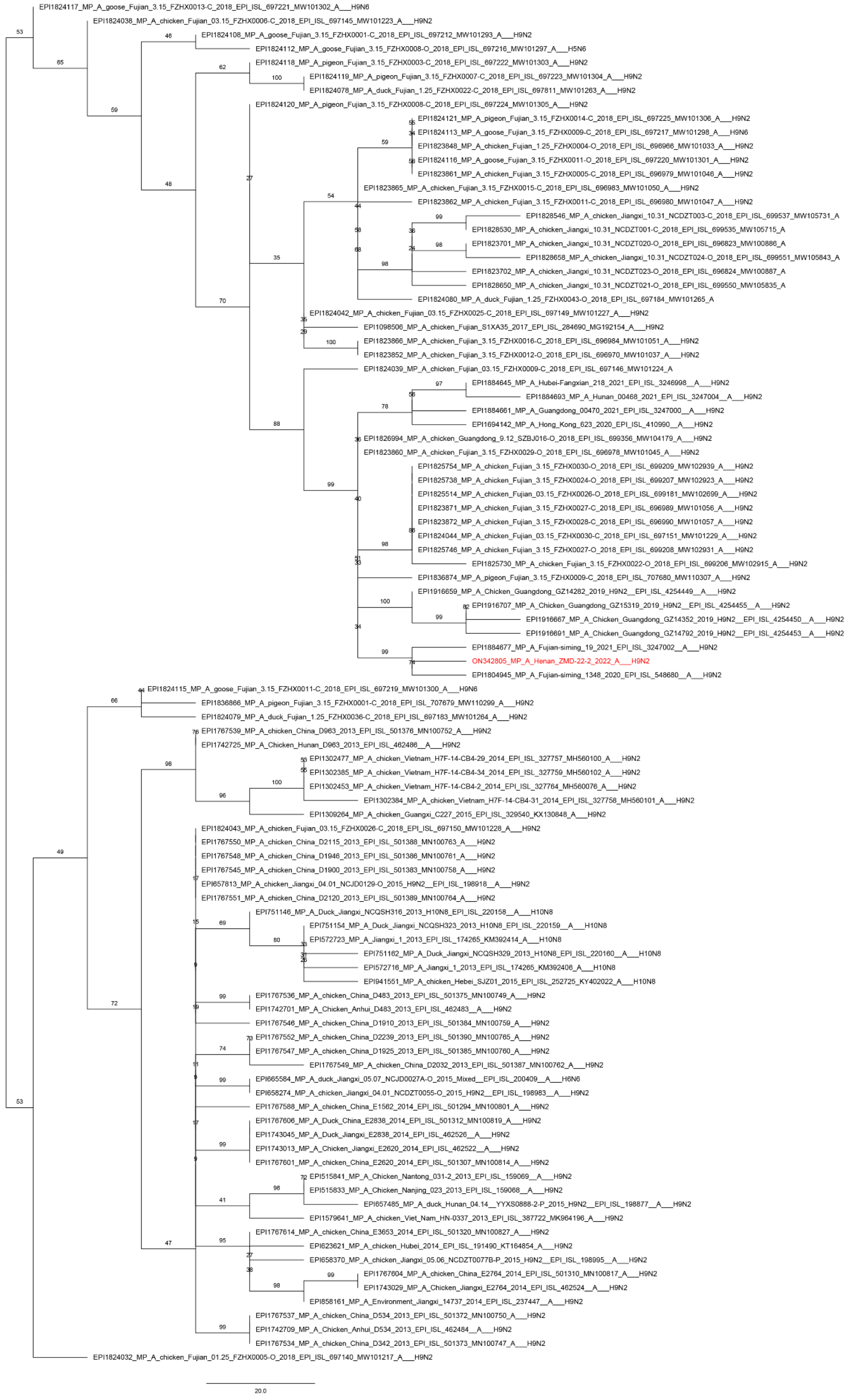

Fig. S3 F(NS)

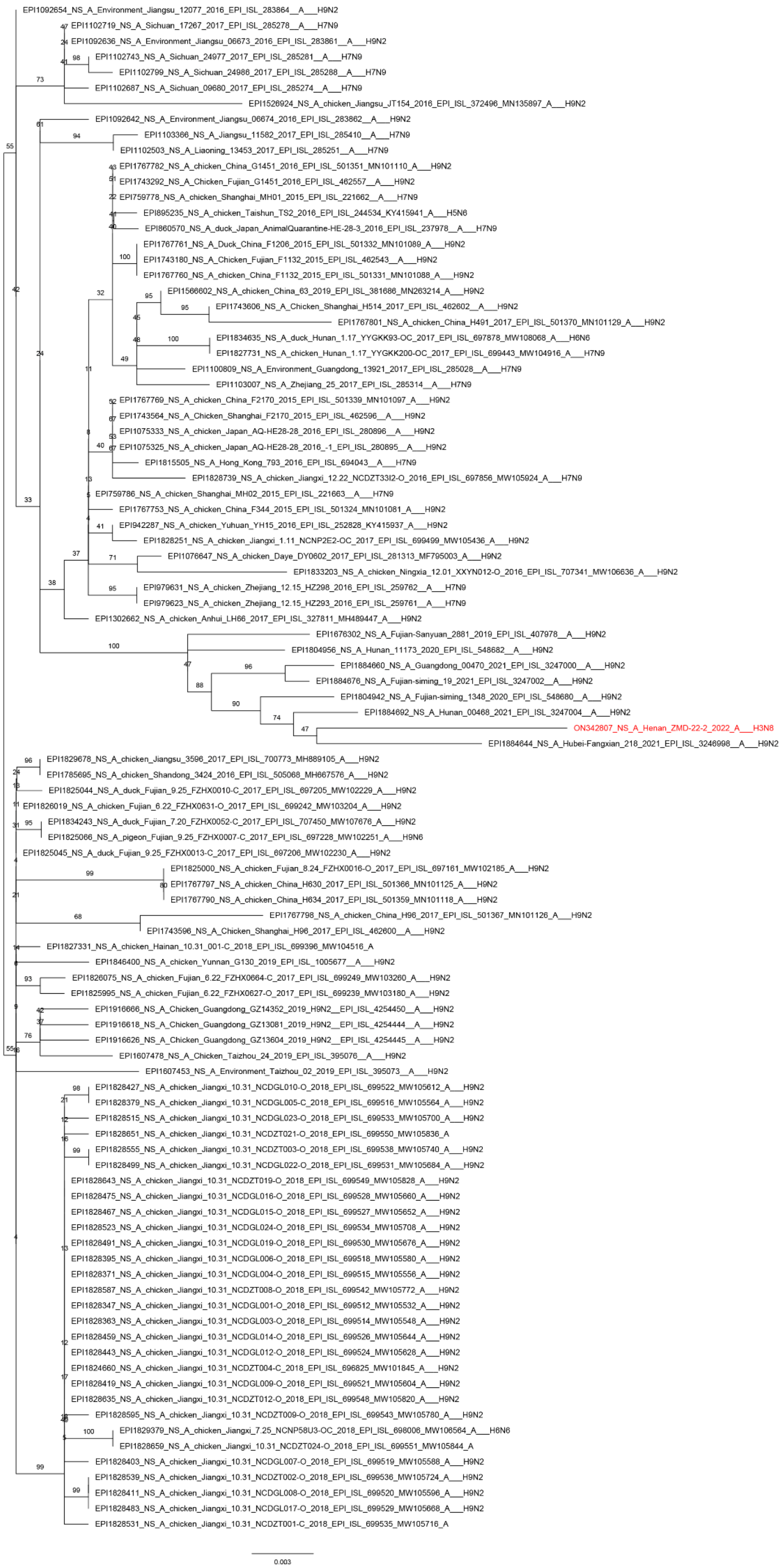

Supplemental Table 1. The selected pathogens tested for the patient.

Pathogens	Methods
Influenza virus A	Real-time RT-PCR and Indirect immunofluorescent assay
Influenza virus B	Real-time RT-PCR and Indirect immunofluorescent assay
Seasonal influenza H3N2 virus	Real-time RT-PCR
Seasonal influenza H1N1 virus	Real-time RT-PCR
2009 pandemic influenza A	Real-time RT-PCR
Respiratory syncytial virus	Real-time RT-PCR, Indirect immunofluorescent assay
H7N9 virus	Real-time RT-PCR
H5N1 virus	Real-time RT-PCR
Human rhinovirus	Real-time RT-PCR
Human parainfluenza virus	Real-time RT-PCR and Indirect immunofluorescent assay
Human adenovirus	Real-time PCR and Indirect immunofluorescent assay
Human coronavirus	Real-time RT-PCR
Human bocavirus	Real-time PCR
Human metapneumovirus	Real-time RT-PCR
Legionella pneumophila	Indirect immunofluorescent assay
Mycoplasma pneumoniae	Indirect immunofluorescent assay
Coxiella burnetii	Indirect immunofluorescent assay
Chlamydia pneumoniae	Indirect immunofluorescent assay

Supplemental Table 2. Primers used for the detection of the current A(H3N8) virus.

Primer	Sequence (5’-3’)
Left	GCAAAAGCAGGGGATACTTTC
Right	CTGATCGTCGGTGATTGTTTT
Probe	FAM-CAGAACCTTCCAGGGAATGA-MGB

Supplemental Table 3. Complications, treatment regimens and duration of the patient.

Signs and treatment	
**Fever**	
Temperature on admission (°C)	39.5
Highest temperature (°C)	41
**Oximetry saturation,** Lowest (%)	55%
**Complications**	
Severe pneumonia	Yes
Liver dysfunction	Yes
Myocardial damage	Yes
Renal damage	Yes
Coagulation dysfunction	Yes
Sepsis	Yes
Systemic inflammatory response syndrome	Yes
**Bacterial co-infection**	Yes
**Mechanical ventilation via endotracheal intubation**	Hospital day 1-44
**Extracorporeal Membrane Oxygenation**	Hospital day 1-24
**Blood transfusion**	
Red blood cells	Hospital day 7, 9, 11, 16 (1U)
Plasma	Hospital day 9 (200mL)
**Glucocorticoids (methylprednisolone)**	Hospital day 1-10 (30mg doses given intravenously, twice a day)
	Hospital day 11-64 (10mg doses given intravenously, twice a day)
**Antibiotic treatment**	
Linezolid	Hospital day 1-2 (75 mL, three times a day)
Meropenem	Hospital day 1-18(0.3g, three times a day)
Compound sulfamethoxazole	Hospital day 2-46 (one tablet, once a day)
**Antiviral treatment**	

Oseltamivir phosphate	Hospital day 1-25 (30mg, twice a day)
Recombinant human interferon α1b	Hospital day 2-25(30ug, twice a day)

Supplemental Table 4. Key molecular characteristics of the A(H3N8) virus identified in this study.

Gene	Mutation	A/H3N8/ZMD-22-2/2022	Mutation comments
**HA**	Cleavage site	PEKQTR/GL	Pathogenic to poultry
	Q226L	Q	RBS position, altered receptor specificity
	G228S	G	
**PB1**	H99Y	H	H5 virus transmissible among ferrets
**PB2**	Q591K	Q	Enhance replication efficiency and Increase virulence in mice
	E627K	K	Increase virulence in mice
	D701N	D	Increase virulence in mice and transmission in mammals
**NP**	N319K	N	Enhance replication efficiency
**M**	N30D	D	Increase virulence in mice
	T215A	A	
**NS**	P42S	S	Increase virulence in mice

Supplemental Table 5. Laboratory test result of dog and cat kept in the house of the patient.

	Dog		Cat	
	**Normal range**	**Value**	**Normal range**	**Value**
Albumin (g/L)	23–40	29.23	22–40	33.94
Alanine aminotransferase (U/L)	10–125	10.76	12–130	46.76
Aspartate transaminase (U/L)	0–50	28	0–48	33
Urea (mmol/L)	2.5–9.6	2.45 (↓)	5.7–12.9	7.25
Cholesterol (mmol/L)	2.84–8.27	3.98	1.68–5.81	3.61
Creatine kinase (U/L)	10–200	323 (↑)	0–314	220
Creatinine (μmol/L)	44–159	34.32 (↓)	71–212	85.88
C-Reactive protein (mg/L)	0.0–10.0	0.21	0.0–10.0	0.34
Glucose (mmol/L)	4.11–7.94	5.62	4.11–8.83	3.79 (↓)
Lactate (U/L)	40–400	388	0–798	147
Protein total (g/L)	52–82	53.56	57–89	67.65
Alkaline phosphatase (U/L)	20-156	116	25-93	31
Triglycerides (mmol/L)	0.56-1.7	0.19 (↓)	0.2-1.1	0.54
Low-density lipoprotein (g/mL)	1.019-1.087	1.29 (↑)	1.030-1.043	0.96
High-density lipoprotein (g/mL)	1.063-1.100	3.23 (↑)	1.063-1.100	2.63 (↑)

Supplemental Table 6. Characteristics of a confirmed patient with A (H3N8) infection and six close contacts.

Characteristics	Patient	Close contacts					
**Relationship**		Grandfather	Grandmother	Father	Aunt	Brother	Sister
**Age (years)**	4	60	60	32	36	8	6
**Sex**	Male	Male	Female	Male	Female	Male	Female
**Occupation**	Children	Farmer	Farmer	Worker	Teacher	Student	Student
**Comorbidity**	None	None	None	None	None	None	None
**History of animal contact**							
Recent visit to poultry market	None	None	None	None	None	None	None
Contact with dog	Frequent	Infrequent	None	None	None	Infrequent	None
Type of contact dog	Daily feeding and playing	Infrequent feeding	-	-	-	Playing	None
Contact with cat	Frequent	Infrequent	None	None	None	Infrequent	None
Type of contact cat	Daily feeding and playing	Infrequent feeding	-	-	-	Playing	None
Contact with chickens	Yes	Yes	Yes	Yes	Yes	Yes	Yes
**Neutralizing antibody titer**	1:121	1:339	1:179	ND*	ND*	Negative	Negative

*The sample was not available and the test was not done.
